# Excited-state symmetry breaking in quadrupolar pull–push–pull molecules: dicyanovinyl *vs.* cyanophenyl acceptors†‡

**DOI:** 10.1039/d3cp02810k

**Published:** 2023-08-02

**Authors:** Pragya Verma, Mariusz Tasior, Palas Roy, Stephen R. Meech, Daniel T. Gryko, Eric Vauthey

**Affiliations:** a Department of Physical Chemistry, University of Geneva, 30 Quai Ernest-Ansermet, CH-1211 Geneva 4 Switzerland eric.vauthey@unige.ch; b Institute of Organic Chemistry, Polish Academy of Sciences 01-224 Warsaw Poland; c School of Chemistry, University of East Anglia, Norwich Research Park Norwich NR4 7TJ UK

## Abstract

A significant number of quadrupolar dyes behave as their dipolar analogues when photoexcited in polar environments. This is due to the occurrence of excited-state symmetry breaking (ES-SB), upon which the electronic excitation, initially distributed over the whole molecule, localises preferentially on one side. Here, we investigate the ES-SB properties of two A–D–A dyes, consisting of a pyrrolo-pyrrole donor (D) and either cyanophenyl or dicyanovinyl acceptors (A). For this, we use time-resolved vibrational spectroscopy, comparing IR absorption and femtosecond stimulated Raman spectroscopies. Although dicyanovinyl is a stronger electron-withdrawing group, ES-SB is not observed with the dicyanovinyl-based dye even in highly polar media, whereas it already takes place in weakly polar solvents with dyes containing cyanophenyl accepting groups. This difference is attributed to the large electronic coupling between the D–A branches in the former dye, whose loss upon symmetry breaking cannot be counterbalanced by a gain in solvation energy. Comparison with analogues of the cyanophenyl-based dye containing different spacers reveals that interbranch coupling does not so much depend on the distance between the D–A subunits than on the nature of the spacer. We show that transient Raman spectra probe different modes of these centrosymmetric molecules but are consistent with the transient IR data. However, lifetime broadening of the Raman bands, probably due to the resonance enhancement, may limit the application of this technique for monitoring ES-SB.

## Abstract 2

Excited-state symmetry breaking is only observed in multipolar dyes with weaker electron accepting groups. This unexpected result can be rationalised in terms of a subtle balance between solvation energy and inter-branch electronic coupling.

## Graphical abstract

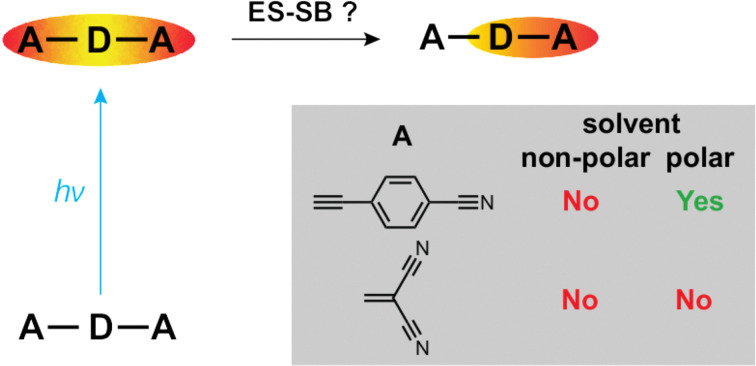

## 1 Introduction

Quadrupolar conjugated molecules consisting of two electron donor–acceptor (D–A) branches arranged in D–A–D or A–D–A motifs are attracting considerable attention for their large two-photon absorption properties that make them promising dyes for a broad range of applications, including fluorescence imaging microscopy,^1–3^ and photopolymerisation.^4–6^ Additionally, A–D–A type molecules are being increasingly investigated as alternative acceptors to fullerenes in organic photovoltaics,^7–9^ as well as emitters in OLEDs.^10–12^

A significant number of these molecules have been found to undergo excited-state symmetry breaking (ES-SB). During this process, the electronic excitation, initially distributed evenly over the whole molecule, localises, at least partially, on one side.^13–15^ The occurrence of this phenomenon was first inferred from the fluorescence solvatochromism of two-branched dyes that were found to be as large as that of the single D–A branch analogues.^13,16–22^ Unambiguous evidence was subsequently obtained from time-resolved IR spectroscopy by monitoring vibrations that are IR silent in the symmetric quadrupolar state and become IR allowed upon ES-SB.^23,24^ Since then, alternative spectroscopic signatures of this process observable by transient electronic absorption or time-resolved fluorescence have also been reported.^25–29^

The results obtained so far revealed the crucial role of the environment, which stabilises the symmetry-broken dipolar state relative to the quadrupolar state and compensates for the loss of coupling between the two branches. Therefore, the occurrence of ES-SB depends on a subtle balance between solvation energy and coupling, which are themselves determined by the nature of the D–A subunits and the linker.

**Fig. 1:**
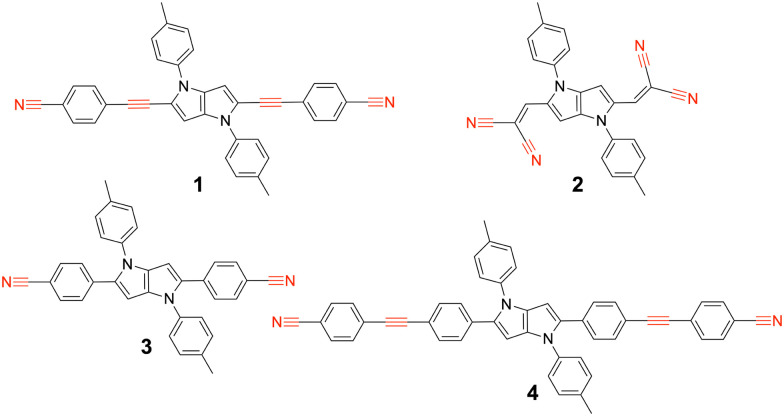
Structure of the A–D–A dyes 1 and 2 studied here and of the related dyes 3 and 4 investigated previously, with the vibrational markers highlighted in red.

We report here on our investigation of the ES-SB properties of two A–D–A dyes based on an electron-rich 1,4-dihydropyrrolo[3,2-*b*]pyrrole donor and two different accepting subunits at positions 2 and 5.^30^ The first one (1, Fig. 1) contains two cyanophenyl acceptors, and is in this respect similar to two other A–D–A dyes investigated previously (3, 4).^31,32^ Like compound 4, this dye contains two vibrational markers, the –C≡N and the –C≡C– stretching modes, allowing for a precise determination of the distribution of the electronic excitation. Comparison of the ES-SB properties of this dye with those of 3 and 4 will give insight into the influence of the D–A distance and the nature of the linker. The second dye, 2, contains dicyanovinyl accepting groups. Although dicyanovinyl is a broadly used electron-withdrawing group in conjugated molecules,^33–37^ ES-SB in dicyanovinyl-based quadrupolar molecules has not been explored so far.

Detection of symmetry breaking is done using time-resolved vibrational spectroscopy. Here, for the first time to the best of our knowledge, we apply both time-resolved IR spectroscopy and femtosecond stimulated Raman spectroscopy (FSRS) to monitor this process.^38,39^ Given that ES-SB in these dyes involves a loss of centrosymmetry, these two techniques are expected to deliver complementary information.

## 2 Experimental

### 2.1 Samples

Dyes 1 and 2 were synthesised and purified as described in ref. 30. The solvents, cyclohexane (CHX), toluene (TOL), chloroform, tetrahydrofuran (THF), dimethylsulfoxide (DMSO) and 2-chloroethanol (2-CE) were of the highest commercially available purity and were used as received. The polyvinylbutyral (PVB, Mowital B60 H) films were prepared by mixing a solution of 2 in dichloromethane to a polymer solution in the same solvent. After the evaporation of the solvent, the films were kept under vacuum for one week. They were then slightly warmed up and compressed between two quartz windows. For the transient absorption experiments, the absorbance was between 0.1 and 0.2 at the excitation wavelength.

### 2.2 Stationary spectroscopy

Electronic absorption spectra were measured using a Cary 50 spectrometer, whereas emission spectra were recorded on a Horiba FluoroMax-4 spectrofluorometer. The fluorescence spectra were corrected using a set of secondary emissive standards.^40^

### 2.3 Time-resolved spectroscopy

#### 2.3.1 Time-resolved fluorescence

Fluorescence lifetime measurements were performed using the time-correlated single-photon counting (TCSPC) technique. Excitation was performed with a pulsed laser diode at 395 nm (Picoquant LDHPC-400b). The instrument response function (IRF) was recorded using a LUDOX solution and the fluorescence dynamics were analysed by iterative reconvolution of exponential functions with the IRF. The full width at half maximum (FWHM) of the IRF was around 200 ps.

#### 2.3.2 Electronic transient absorption (TA) spectroscopy

Electronic transient absorption (TA) measurements were performed with a setup described in ref. 41 and based on an amplified Ti:Sapphire system (Solstice Ace, Spectra-Physics), producing 35 fs pulses centred at 800 nm with a 5 kHz repetition rate. The pump pulses were produced either by frequency doubling a fraction of the amplifier output or using a TOPAS-Prime combined with a NirUVis module (Light Conversion). The pump intensity on the sample was between 0.15 and 0.75 mJ cm^−2^. Probing was achieved from about 320 to 750 nm using white light pulses generated in a 3 mm CaF_2_ plate. The polarisation of the pump pulses was at the magic angle with respect to that of the probe pulses. The sample cell was 1 mm thick and the IRF had a FWHM varying between 80 and 350 fs, depending on the probe wavelength.

#### 2.3.3 Time-resolved infrared spectroscopy

The time-resolved infrared spectroscopy measurements were performed using a setup described in detail previously,^42^ and based on an amplified Ti:Sapphire system (Spectra-Physics, Solstice, 100 fs, 800 nm, 1 kHz). The pump pulses were generated from the second harmonic of the output of an optical parametric amplifier (Light Conversion, TOPAS-C). Probing was achieved using the output of an optical parametric amplifier (TOPAS C, Light Conversion) combined with a non-collinear difference-frequency-mixing module (NDFG, Light Conversion). These pulses were dispersed in a Triax 190 spectrograph (Horiba, 150 lines per mm) and detected with a 2 × 64 elements MCT array (Infrared Systems Development). The samples were flowed through a cell with CaF_2_ windows and a 500 μm spacer and had an absorbance of less than 0.3 at the excitation wavelength.

#### 2.3.4 Femtosecond stimulated Raman spectroscopy (FSRS)

The FSRS data were measured using a spectrometer described in detail elsewhere.^43^ FSRS measurements comprise a combination of a pump pulse with a pair of probe pulses, a narrowband picosecond pulse and a broadband ultrafast white light continuum pulse. The pump at 430 nm was obtained from a Light conversion OPA TOPAS prime. The Raman pump at 620 nm, resonant with the transient absorption, was from a light conversion TOPAS ps OPA pumped at 400 nm by a second harmonic bandwidth compressor. The continuum was generated in a sapphire plate by focusing the 1250 nm output of a second TOPAS prime. All OPAs were pumped by a Spectra Physics Spitfire ACE operating at 1 kHz. The overall time resolution was 100 fs. Spectra were measured with a spectrometer and CCD combination, and the overall spectral resolution was 10 cm^−1^. The raw FSRS data were collected, processed and background corrected as described in detail in the ESI‡ (Section S5).

### 2.4 Quantum-chemical calculations

All calculations were carried out in the gas phase at the density functional theory (DFT) or time-dependent (TD) DFT level using the CAM-B3LYP functional^44^ combined with the 6-31G(d,p) basis set, as implemented in the Gaussian 16 (rev.B) package.^45^ Addition of a 1 GV m^−1^ dipolar electric field was done using the ‘field = *x* + 20’ keyword, where *x* is the long molecular axis. All vibration frequencies were corrected by a factor of 0.93.

## 3 Results

### 3.1 Stationary spectroscopy

**Fig. 2:**
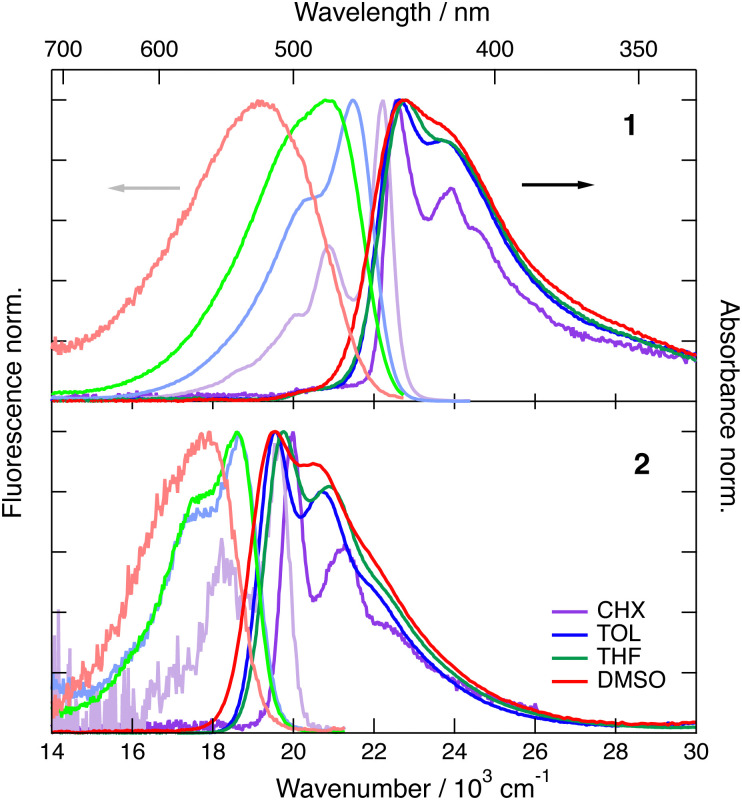
Stationary electronic absorption and emission spectra recorded with dye 1 (top) and dye 2 (bottom) in various solvents.

As illustrated in Fig. 2, the S_1_–S_0_ absorption and emission bands of 1 and 2 exhibit a distinct vibronic structure in the non-polar CHX. Upon increasing the solvent polarity, the band broadens and the vibronic structure becomes hardly visible, especially in the fluorescence spectra. The absorption band of both dyes presents only small solvatochromism, which seems to correlate better with *f*(*n*^2^) than with Δ*f* = *f*(*ε*) − *f*(*n*^2^), where *f*(*x*) = 2(*x* − 1)/(2*x* + 1), *n* and *ε* being the refractive index and static dielectric constant of the solvent, respectively (Fig. S1, ESI‡). This suggests that the absorption solvatochromism is mostly due to dispersion interactions.^46^ By contrast, the fluorescence band maximum shows a dependence on the orientational polarisation function, Δ*f*, that is larger for 1 by a factor of about 2.5 compared to 2 (Fig. S2, ESI‡).

The magnitude of the emission solvatochromism of 1 is between those found earlier with 3 and 4, for which ES-SB was observed.^24,32^ Based on this, the solvent dependence of the absorption and emission spectra of 1 is consistent with the symmetric and quadrupolar ground and Franck–Condon excited states and with a dipolar relaxed excited state. The emission solvatochromism of 2 is smaller by a factor of about 1.5 than that observed with 3. Considering that quadrupole-dipole interactions also result in spectral shifts scaling with Δ*f*,^46^ no unambiguous conclusion on the occurrence of ES-SB with 2 can be drawn.

### 3.2 Electronic transient absorption spectroscopy

**Fig. 3:**
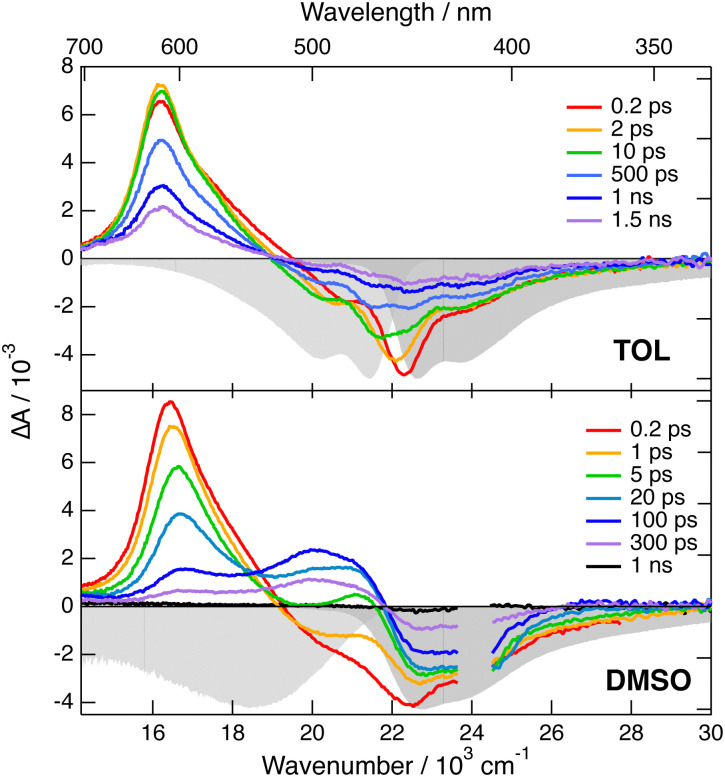
Transient electronic absorption spectra recorded at various time delays after 400 nm excitation of dye 1 in toluene (top) and DMSO (bottom). The negative stationary absorption and stimulated emission spectra are shown in grey.

**Fig. 4:**
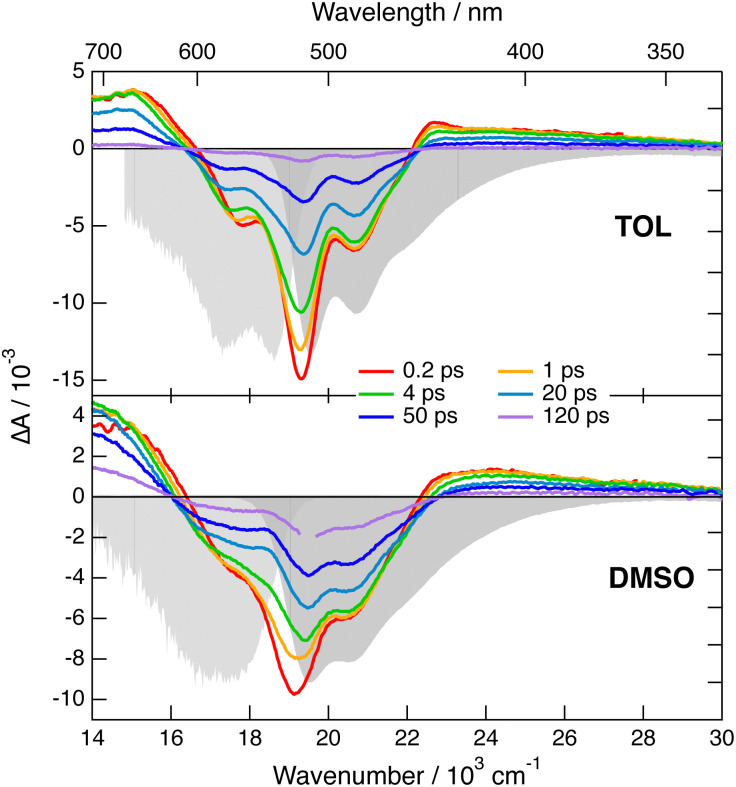
Transient electronic absorption spectra recorded at various time delays after 510 nm excitation of dye 2 in toluene (top) and DMSO (bottom). The negative stationary absorption and stimulated emission spectra are shown in grey.

Electronic transient absorption (TA) spectra recorded with both dyes in TOL and DMSO are presented in Fig. 3 and 4, and those measured in other solvents are shown in Fig. S4 and S6 (ESI‡). The TA data were analysed globally assuming a series of successive exponential steps with increasing lifetime.^47,48^ The resulting evolution-associated difference spectra (EADS) and time constants are depicted in Fig. S5 and S7 (ESI‡). The transient spectra measured with 1 are dominated by a positive band centred just above 600 nm and by a negative band located below 500 nm, which can be attributed to stimulated emission (SE) and ground-state bleach (GSB). In CHX, these features decay with a ∼1 ns time constant without significant spectral dynamics. As this timescale matches the fluorescence lifetime measured by TCSPC, the positive band is assigned to S_*n>*1_ ← S_1_ excited-state absorption (ESA). In agreement with the fluorescence lifetime measurements (Fig. S3, ESI‡), the decay of the ESA and SE bands accelerates significantly upon increasing the solvent polarity. Additionally, the SE band undergoes a red shift during the first few tens of ps with an amplitude that increases with the solvent polarity. These dynamics can be attributed to the time-dependent Stokes shift that results from the equilibration of the solvent surrounding the excited molecules.^49^ Whereas the SE decays completely within the experimental time window, the GSB decays only partially to a residual amplitude that decreases with increasing solvent polarity. The residual spectrum comprises a positive band above 600 nm that is distinct from the S_*n*>1_ ← S_1_ ESA (Fig. S5, ESI‡). This late transient spectrum is attributed to the lowest triplet excited state of 1 populated upon intersystem crossing (ISC). This assignment is consistent with a previous observation of the triplet state of 3, the short-branch analogue of 1, in CS_2_, where ISC is accelerated by the external heavy atom effect.^31^ The T_*n*>1_ ← T_1_ band of 3 was found to be slightly red shifted relative to the S_*n*>1_ ← S_1_ band, as observed here with 1. By comparing the amplitude of the GSB in the first and last EADS, triplet yields of the order of 0.2 and 0.04 can be estimated in TOL and DMSO, respectively. These values correspond to an ISC time constant of about 5 ns. The solvent polarity dependence of the singlet excited state lifetime, which decreases from 1.3 ns to 240 ps when going from CHX to DMSO (Fig. S5, ESI‡), can be explained by an acceleration of the internal conversion due to the decrease of the S_1_–S_0_ gap,^50^ and the change in the nature of the S_1_ state. Similar strong solvent dependence of the excited-state lifetime was already observed with other multi-branched D–A molecules.^23,51–54^

The TA spectra measured with 2 exhibit a negative band due to SE and GSB, as well as weaker positive features above 600 nm and below 450 nm that can be attributed to S_*n*>1_ ← S_1_ ESA (Fig. 4 and Fig. S6, ESI‡). Like with dye 1, the SE band exhibits a red shift during the first tens of ps. However, its amplitude is much smaller than with 1, in agreement with the weaker fluorescence solvatochromism. Subsequently, all of these bands decay rapidly to give a residual spectrum with two very weak bands: a negative one in the GSB region and a positive one around 550 nm (Fig. S7, ESI‡). This residual spectrum, whose amplitude is the largest in DMSO, is tentatively attributed to the lowest triplet state of 2. From the amplitude of the last EADS in the GSB region, the triplet yield in DMSO is estimated to be of the order of 0.01. These results point to a markedly shorter excited-state lifetime of 2 compared to 1. Contrary to 1, this lifetime, amounting to 46, 35 and 88 ps in TOL, THF and DMSO, respectively, does not correlate with the solvent polarity. However, it rather seems to depend on the solvent viscosity, suggesting that a non-radiative transition involving large amplitude motion could control the decay of the excited state of 2.^55,56^ To check this, we repeated the TA measurements with 2 in a rigid PVB film. As illustrated in Fig. S6 (ESI‡), the TA spectra are similar to those in TOL, but the dynamics are significantly slower. According to the global analysis, the decay of the S_1_ state population is mostly associated with a 370 ps time constant (Fig. S7, ESI‡). Additionally, the amplitude of the residual spectrum assigned to the triplet state is significantly larger than in liquid solutions and points to a triplet yield of about 0.08. These results confirm that the fast excited-state decay of 2 is due to a non-radiative transition involving large amplitude motion. Identification of this mode is out of the scope of the present investigation.

Whereas these TA measurements in the visible region give precious information on the excited-state dynamics of both dyes, they do not give any detail on the distribution of the excitation and on the possible occurrence of ES-SB. For this, we turn to time-resolved vibrational spectroscopy.

### 3.3 Time-resolved vibrational spectroscopy

**Fig. 5:**
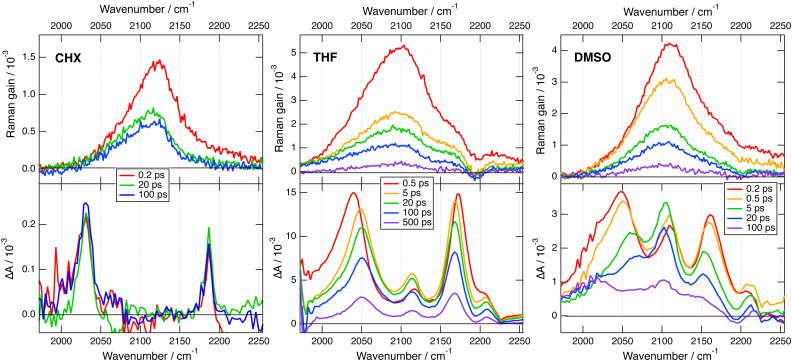
Transient stimulated Raman spectra (top) and transient IR absorption spectra (bottom) recorded at various time delays after 430 nm excitation of dye 1 in various solvents. The FSRS measurements were done in a 1 : 15 TOL/CHX (v/v) mixture and not in pure CHX.

Transient IR and stimulated Raman spectra in the –C≡C– and –C≡N stretching region measured with 1 in CHX, THF and DMSO are presented in Fig. 5, whereas transient IR spectra measured in TOL and CHCl_3_ together with the EADS obtained from global analysis are shown in Fig. S9 and S10 (ESI‡). Because of the limited solubility of 1 in CHX, the signal to noise ratio is weak but sufficient to detect two positive bands at 2031 and 2187 cm^−1^. By comparison the stationary IR absorption spectrum measured in this region with 1 in CHCl_3_ exhibits a broad band at 2190 cm^−1^ as well as a narrower and less intense one at 2230 cm^−1^ (Fig. S8, ESI‡). The transient IR spectra show negligible dynamics apart from a concurrent decay of both bands on the ns timescale. To reach a sufficient concentration of 1, the FSRS measurements were performed in a 1 : 15 TOL/CHX (v/v) mixture. The transient Raman spectra exhibit a broad band at 2123 cm^−1^ with a possible shoulder around 2200 cm^−1^. This band undergoes a partial decay within a few ps followed by a slower one on the nanosecond time scale, similar to the transient IR bands. Because of the resonant character of the Raman spectrum, the time dependence of Raman intensity does not necessarily reflect the population dynamics and will, therefore, not be considered further. No band at the frequencies found in the transient IR spectra can be observed. Similarly, the Raman band has no corresponding band in the IR spectra. These results are consistent with the exclusion principle for the vibrations of centro-symmetric molecules. This is clear evidence of the symmetric and quadrupolar nature of the S_1_ state of 1 in a non-polar environment. The 2031 and 2187 cm^−1^ IR bands are assigned to the antisymmetric –C≡C– and –C≡N stretching vibrations of 1 in the quadrupolar S_1_ state, whereas the 2123 cm^−1^ Raman band is attributed to the symmetric –C≡C– stretch. This interpretation is supported by quantum-chemical calculations at the (TD) DFT level (CAM-B3LYP/6-31G(d,p)) (Table S1, ESI‡), which predict a symmetric and quadrupolar S_1_ state with one –C≡C– and one –C≡N IR band associated with the antisymmetric stretching modes downshifted by about −200 and −25 cm^−1^ with respect to the same vibrations in the ground state. The symmetric –C≡C– stretching band is predicted to be located between the two IR bands, as found experimentally. Furthermore, the symmetric –C≡N stretching mode is calculated at 2220 cm^−1^, where the shoulder in the Raman spectrum is observed.

The transient IR spectra in weak to medium polar solvents, TOL, CHCl_3_ and THF, present considerably more complex dynamics. At early times, they consist of essentially the same two bands observed in CHX. Within a few ps, the –C≡C– band shifts to higher frequency by ∼12 cm^−1^, the –C≡N band moves to lower frequency by a few cm^−1^, and two weak bands appear around 2115 and 2210 cm^−1^. Afterwards, the spectral shape remains essentially unchanged and all bands decay on the hundreds of ps timescale. The early Raman spectra in THF are dominated by a single broad band centred at 2110 cm^−1^ with a shoulder around 2210 cm^−1^. During the first few ps, this band decays partially and shifts to lower frequency by approximately −15 cm^−1^, before decaying completely on the hundreds of ps timescale. The IR spectral dynamics can be attributed to ES-SB. At early times, the S_1_ state of 1 is symmetric as in CHX and, within a few ps, it acquires some dipolar character as the electronic excitation becomes more localised on one of the two branches. Because of the loss of centro-symmetry, the exclusion principle does not hold anymore, and the symmetric –C≡C– and –C≡N stretches are no longer IR forbidden. This is the reason why the two weak bands at 2115 and 2210 cm^−1^, which can be assigned to symmetric stretching modes, becomes visible in the transient IR spectrum. The timescales on which these changes take place are of the same order of magnitude as those reported for solvent motion,^57^ in agreement with previous studies.^23,24,31^ This is consistent with the fact that the symmetry-broken state is stabilised relatively to the quadrupolar state by a gain in solvation energy.

All four bands are already visible in the earliest transient IR spectra measured in the highly polar DMSO. Within a few ps, the antisymmetric –C≡C– band decreases and shifts to higher frequency by ∼20 cm^−1^, the antisymmetric –C≡N band shifts to lower frequency by ∼−6 cm^−1^, and the symmetric –C≡C– band rises and becomes dominant. Afterward, all bands decay with a 250 ps time constant. The Raman spectra in DMSO consist of a single broad band initially centred at 2110 cm^−1^. This band undergoes a frequency down-shift of ∼−5 cm^−1^ during the first few ps, before decaying almost entirely within 500 ps the upper time limit of the experiment. The IR data indicate that ES-SB in DMSO occurs within the instrument response. Such ultrafast ES-SB was already reported with dyes 3 and 4 and was explained by the contribution of inertial solvent motion, which brings sufficient gain in solvation energy to induce partial ES-SB.^24,58^ However, the subsequent spectral dynamics points to a further increase of the extent of symmetry breaking on the timescale of diffusive solvent motion.

To obtain theoretical support on the effect of ES-SB on the frequencies of these vibrational modes, TD-DFT calculations were also performed with a dipolar analogue of 1 and 1d (Fig. S14, ESI‡), with one cyano group replaced by a hydrogen atom. These calculations indicate that, as expected, the S_1_ state of 1d has a charge-transfer character with a higher excited electron density on the branch with the cyano end group (Fig. S14, ESI‡). According to these calculations, going from the quadrupolar excited state of 1 to the dipolar excited state of 1d results in a 14 cm^−1^ frequency up-shift of the antisymmetric –C≡C– band and to a significant oscillator strength for IR absorption of the symmetric –C≡C– stretch (Table S1, ESI‡). This is in excellent agreement with the observed transient IR dynamics. These calculations also predict that both symmetric and antisymmetric –C≡C– stretches becomes Raman active. Unfortunately, the transient Raman bands are too broad to resolve the symmetric and antisymmetric –C≡C– stretching modes. However, the observed frequency down-shift of the Raman band can be accounted for by the rise of the antisymmetric band at lower frequency and the parallel intensity decrease and shift of the symmetric band upon ES-SB (Table S1, ESI‡).

In summary, these transient vibrational spectroscopy data reveal that ES-SB is not taking place with dye 1 in non polar solvents. However, it becomes operative already in weakly polar media like toluene, and its extent increases with solvent polarity. Nevertheless, full localisation of the excitation on a single branch does not take place even in highly polar solvent. If this were the case, only one –C≡C– band and one –C≡N band should be visible in the transient IR spectra.

Dye 2 has four –C≡N stretching vibrations. According to ground-state DFT calculations, only the two anti-symmetric modes are IR active with a frequency difference of 8 cm^−1^ (Table S2, ESI‡). However, the higher-frequency band, where the two –C≡N on each dicyanovinyl group oscillate in phase, is >10 times more intense than the other. This result is consistent with the stationary IR absorption spectrum of 2 measured in CHCl_3_, that shows a single –C≡N stretching band at 2220 cm^−1^ (Fig. S8, ESI‡).

**Fig. 6:**
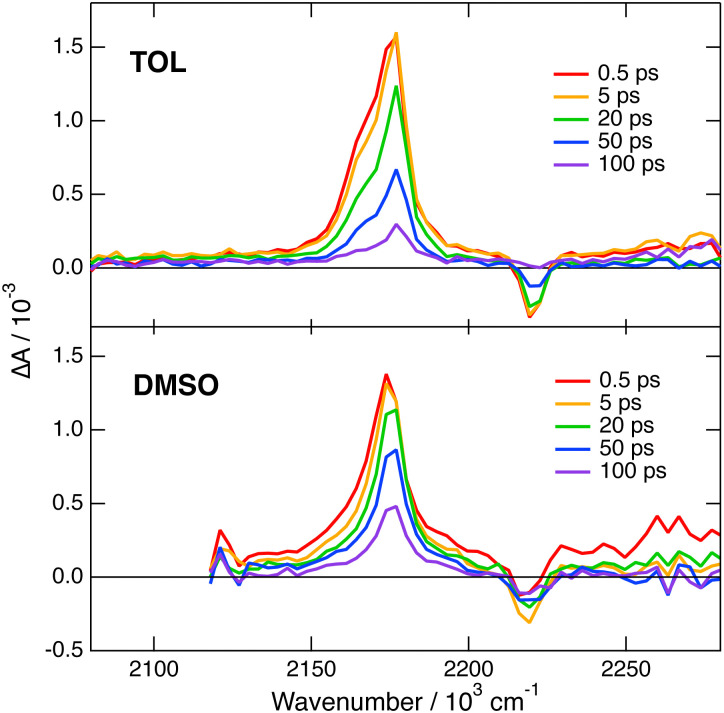
Transient IR absorption spectra recorded at various time delays after 480 nm excitation of dye 2 in toluene (top) and DMSO (bottom).

Transient IR measurements with 2 in the –C≡N stretching region were performed in TOL, THF, DMSO and 2-chloroethanol (2-CE). The latter solvent was selected for its highly protic character^59^ to look for a possible enhancement of ES-SB upon hydrogen-bond interactions with the cyano groups. Transient IR spectra in TOL and DMSO are presented in Fig. 6, whereas those in the other solvents as well as the results from global analysis are shown in Fig. S11 and S12 (ESI‡). In contrast with 1, the transient IR spectra exhibit negligible solvent dependence. In all solvents, the spectra consist of a negative band around 2220 cm^−1^, which can be assigned to the bleaching of the ground-state population, and a positive band initially around 2174 cm^−1^ with a low frequency shoulder in TOL and THF. This band narrows and shifts to higher frequency by 3–4 cm^−1^ within the first 10 ps. Afterwards, both bands decay to zero in a few tens of ps, in agreement with the excited-state lifetime deduced from the electronic TA measurements. No residual spectrum that could be attributed to the triplet state could be detected. The triplet population observed in the electronic TA measurements is probably too small to be resolved here.

This invariance to the solvent of the transient IR spectrum of 2 contrasts with the results obtained with 1 and suggests that ES-SB is not taking place with this dye. However, to ascertain this conclusion, the effect of ES-SB on the vibrational spectrum of an A–D–A molecule with dicyanovinyl acceptors should be known. To this aim, we performed gas-phase TD-DFT calculations (CAM-B3LYP/6-31G(d,p)) of 2 and of a dipolar analogue, 2d (Fig. S15, ESI‡), with the two –C≡N of one dicyanovinyl group replaced by methyl groups. The calculations with 2 predict a symmetric and quadrupolar S_1_ state and two IR absorption bands due to the antisymmetric –C≡N stretching modes: an intense one at 2202 cm^−1^ due to the in-phase –C≡N stretching mode and a three times less intense one at 2185 cm^−1^ due to the out-of-phase mode (Table S2, ESI‡). This result is qualitatively consistent with the experimental data in TOL and THF, with the shoulder down-shifted by about −13 cm^−1^ relatively to the main band. The same calculations with the analogue 2d points to a dipolar S_1_ state with a higher excited electron density on the branch with the dicyanovinyl acceptor (Fig. S15, ESI‡). They predict two IR bands, one due to the in-phase stretching at 2181 cm^−1^, and the other, smaller by a factor of 1.7 at 2146 cm^−1^ (Table S2, ESI‡). Therefore, according to these results, the weaker IR band responsible for the shoulder should move further away from the other band and its relative intensity should increase upon ES-SB. Of course, these calculations with 2d mostly account for the effect of full localisation of the excitation and not really for a partial symmetry breaking. To have a better idea on how a partial ES-SB would affect the IR spectrum, we repeated the calculations with 2 in the S_1_ state in the presence of an electric dipole field of 1 GV m^−1^ oriented along the long molecular axis. In such a field, the S_1_ state of 2 has a dipole moment of 4.4 D pointing along the long molecular axis. Moreover, all four –C≡N stretching vibrations become IR active and located within 40 cm^−1^ (Table S2, ESI‡). Consequently, partial ES-SB should lead to a strong broadening of the IR band observed in the quadrupolar state. Such an effect is not found experimentally.

Based on these results and in agreement with the small fluorescence solvatochromism, one can conclude that no significant ES-SB is taking place with 2 even in a highly polar solvent like DMSO or a highly protic solvent like 2CE.

## 4 Discussion

The above-described data reveal that ES-SB is taking place with dye 1 already in weakly polar solvents, whereas it is not operative with dye 2 even in highly polar environments. This result is *a priori* surprising given that dicyanovinyl is a stronger electron attracting group than cyanophenyl. Indeed, the reduction potential of dicyanovinyl substituted systems is typically in the −1.8 to −1.6 V *vs.* SCE range,^36,60^ and that of benzonitrile amounts to −2.3 V *vs.* SCE.^61^ Consequently, charge transfer in 2 should be in principle favoured compared to 1. However, the electron donating and accepting strength of the constituents of a quadrupolar molecule is not the only parameter controlling ES-SB.

**Fig. 7:**
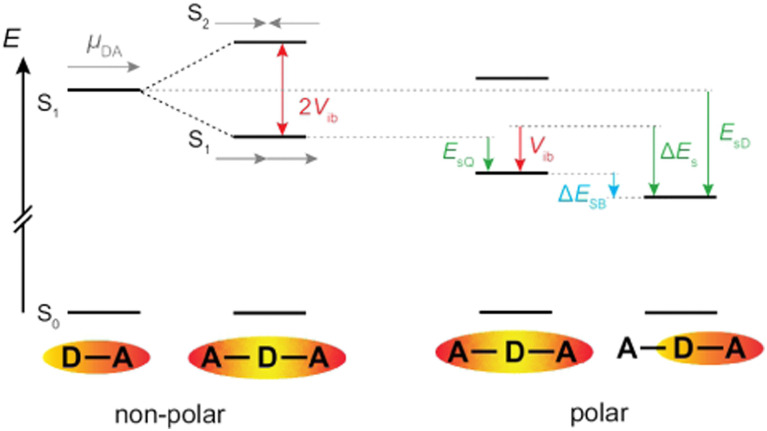
Left: Energy levels of an A–D–A dye based on the Kasha excitonic model assuming that each branch corresponds to a D–A chromophore. Right: Effect of dipolar solvation on the energy of the quadrupolar and the symmetry-broken excited states.

Fig. 7 illustrates a typical energy-level scheme of an A–D–A molecule based on the Kasha's excitonic model.^62,63^ Each D–A branch is a chromophoric unit characterised by a charge-transfer electronic transition with a dipole, *μ⃗*_DA_, aligned along the D–A axis. According to this model, the two lowest excited states of the quadrupolar A–D–A are split by 2*V*_ib_, where *V*_ib_ is the interbranch coupling, which arises from dipole–dipole interactions. The lower excitonic state results from the additive combination of the transition dipoles of each individual D–A branch, whereas the upper state arises from their subtractive combination. Consequently, the S_1_ ← S_0_ transition is one-photon allowed and two-photon forbidden, while the opposite selection rules hold for the S_2_ ← S_0_ transition.^64,65^ Upon full symmetry breaking, the excitation localises on one D–A branch leading to a loss of the interbranch coupling energy, *V*_ib_. For ES-SB to be operative, this loss should be compensated by a gain in energy of the symmetry-broken state. This energy gain can arise from structural changes and/or solvation.^66^ Substantial structural stabilisation due to an alkyne to allene isomerisation was observed in a three-branched octupolar molecule.^53^ However, this energy was not sufficient to break the symmetry in an apolar medium. In all cases reported so far, ES-SB was found to take place in polar environments only,^17,23,24,26,31,52–54,66–74^ suggesting that it is mainly due to the solvation energy gained upon going from a quadrupolar to a dipolar state:

1 Δ*E*_s_ = *E*_s,D_ − *E*_s,Q_,

where *E*_s,D_ and *E*_s,Q_ are the dipolar solvation energies of the dipolar and quadrupolar excitation states (Fig. 7).

Consequently, the energy difference between the quadrupolar excited state and the purely dipolar state, with the excitation entirely localised on one branch is:^71^

2 Δ*E*_SB_ ≃ Δ*E*_s_ − *V*_ib_,

As illustrated in Fig. 7, the interbranch coupling energy, *V*_ib_, can be estimated from the Davidov splitting. In practice, this can be done by taking the energy difference between the lowest energy band in the one-photon (S_1_ ← S_0_) and two-photon (S_2_ ← S_0_) absorption spectra. Using the two-photon absorption spectra reported in ref. 30, *V*_ib_ values of 2530 and 5740 cm^−1^ are found for 1 and 2, respectively (Table 1).

**Table 1:** Interbranch coupling energy, *V*_ib_, determined experimentally and from quantum-chemical calculations, dipolar solvation energy, Δ*E*_fl_/2, and degree of asymmetry, *D*, in DMSO determined from eqn (5)

Dye	1	2	3^a^	4^b^
*V*_ib_(exp)/cm^−1^	2530	5740	1800	1600
*V*_ib_(QM)/cm^−1^	3300	5040	2540	1900
−Δ*E*_fl_/2 cm^−1^	1500	550	1060	2600
*D*	0.8	0.4	0.8	0.95

a From ref. 24.

b From ref. 32.

Alternatively, *V*_ib_ can be obtained from TD-DFT calculations after proper identification of the two excitonic states. They can be recognised by a large and a zero dipole for one-photon absorption from the ground state. Additionally, their wavefunctions should have opposite parity. For example, the S_1_ ← S_0_ and S_2_ ← S_0_ transitions of 1 are mostly due to a HOMO–LUMO and a HOMO–LUMO+1 one-electron transition, respectively. As shown in Fig. S16 (ESI‡), the LUMO and LUMO+1 of 1 have similar shapes but opposite parity. In the case of 2, the upper excitonic state is the S_3_ state. According to this procedure, *V*_ib_ amounts to 3300 and 5040 cm^−1^ for 1 and 2, respectively (Fig. S16, ESI‡). Although these values are not identical to those obtained experimentally (Table 1), they are in qualitative agreement and confirm the markedly larger interbranch coupling in 2. This difference can be accounted for by the shorter branches in 2, the distance between two terminal N atoms along the long axis amounting to 2.3 and 1.4 nm for 1 and 2, respectively.

The solvation energy gained upon full ES-SB is less straightforward to estimate because the dipolar solvation energy of a quadrupolar molecule is not well accounted for by the standard expression for point quadrupole–point dipole interaction.^46^ Previous investigations of the solvatochromism of multipolar molecules suggest that the solvation energy calculated from this expression is strongly underestimated.^53^ A more realistic approach is to consider the quadrupole as two oppositely oriented point dipoles separated by the interbranch center-to-center distance. Within this approximation, the dipolar solvation energy of the quadupolar state, *E*_s,Q_, amounts to approximately a third of that of the purely dipolar state, *E*_s,D_/3.^71^ Based on this and eqn (2), the condition for full ES-SB is:3
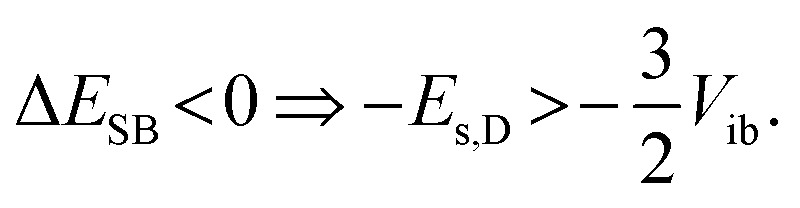


Consequently, for full symmetry breaking to take place in 1 and 2, the solvation energy of the dipolar excited state should exceed −3800 and −8500 cm^−1^, respectively.

The dipolar solvation energy in the excited state in a given solvent corresponds to one half of the fluorescence shift measured by going from the apolar CHX to this solvent, Δ*E*_fl_. Accordingly, the dipolar solvation energy of 1 and 2 in the S_1_ state in DMSO amounts to about −1500 and −550 cm^−1^, corresponding to 0.6*V*_ib_ and 0.15*V*_ib_. The associated gain in solvation energy, Δ*E*_s_, is clearly not sufficient to stabilise a fully symmetry-broken state. In the case of 2, the solvation energy in DMSO is small enough to be due to the solvation of the quadrupolar state, indicative of an absence of ES-SB. For 1, the gain in solvation energy can only counterbalance a partial decrease of *V*_ib_. Therefore, ES-SB is not complete and the electronic excitation is still delocalised on both D–A branches, but unevenly. This agrees very well with the experiments, which point to partial ES-SB with 1 and to an essentially quadrupolar excited state of 2 in DMSO. For 1, partial ES-SB is already observed in TOL but is markedly smaller than in DMSO. Occurrence of ES-SB in TOL most probably arises from the quadrupolar rather than from the dipolar properties of this solvent.^31^

The extent of symmetry breaking can be estimated using the theoretical model developed by Ivanov,^75–78^ where the most relevant parameters are *V*_ib_, the solvation energy and the Coulombic interactions arising from the negative charges on the left and right A subunits, *δ*_L,R_ = *e*|*a*_L,R_|^2^, with |*a*_L_|^2^ + |*a*_R_|^2^ = 1. The degree of asymmetry, *D*, is defined as:^75^

4 *D* = |*a*_L_^2^ − *a*_R_^2^|,

and can be estimated as:5
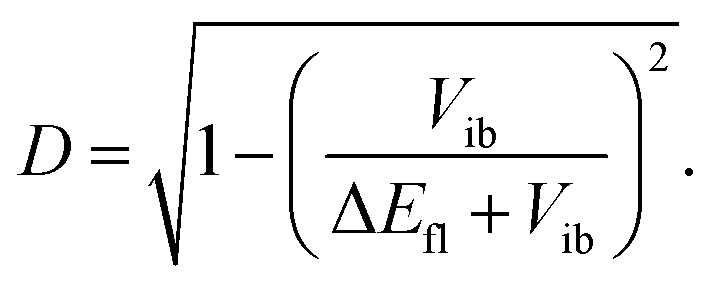
Assuming that only 2/3 of Δ*E*_fl_ are due to dipole–dipole interaction, this model predicts *D* values of 0.8 and 0.4 for 1 and 2 in DMSO. The value for 2 seems quite large in view of the experimental results. However, *D* as calculated from eqn (5) varies strongly with Δ*E*_fl_ when |Δ*E*_fl_| ≪ |*V*_ib_|. Therefore, the error on this value is particularly large for 2. Despite this, the decrease of *D* upon going from dye 1 to dye 2 predicted by the model is consistent with the experimental results.

Now we compare the ES-SB properties of 1 with those of dyes 3 and 4, which also contain cyanophenyl acceptors and only differ by the length of the branches. The distance between the two terminal N atoms increases from 1.8, to 2.3 and 3.2 nm when going from 3 to 1 and 4. Despite different branch lengths, dye 1 and 3 exhibit very similar behaviour, namely ES-SB is observed in weakly polar solvents and in quadrupolar solvents like TOL and remains only partial even in highly polar and protic environments.^24,31^ The solvation energy of 3 in the S_1_ state in DMSO amounts to 0.6*V*_ib_ like for 1. Consequently, the degree of asymmetry, *D*, in this solvent is also equal to 0.8. Contrary to what could be expected for a A–D–A with shorter branches, the interbranch coupling, *V*_ib_, determined from the absorption spectra is smaller than for 1, namely 1800 *vs.* 2530 cm^−1^. The same trend is found with the *V*_ib_ values obtained from quantum-chemical calculations (Table 1). This larger *V*_ib_ in 1 can be explained by the presence of the ethynyl groups that enhances conjugation.

The branches of dye 4 comprise an ethynyl group like those of 1, and contain additionally a benzene ring. The transient IR spectra of 4 in CHX did not exhibit any –C≡N bands, contrary to those of 1 (Fig. 5).^32^ This result was explained by a distribution of the S_1_ excitation that does not extend to the two CN capping groups. In highly polar solvents, the transient IR spectra of 4 consisted of only one –C≡C– and one –C≡N stretching band, indicative of full symmetry breaking. This outcome can be attributed to two factors. First, the permanent electric dipole moment of the symmetry-broken state of 4 is larger than that of 1 and 3 because of the longer branches. This leads to a larger solvation energy, in polar media, with Δ*E*_fl_/2 = 2600 cm^−1^.^32^ Second, the interbranch coupling is significantly smaller than that of 1, namely 1600 *vs.* 2500 cm^−1^, in agreement with the presence of an additional benzene ring in the arms. Consequently, the solvation energy of 4 in the S_1_ state amounts to 1.6*V*_ib_ in highly polar media, fulfilling the condition for complete ES-SB expressed by eqn (3).

Finally, we compare the performance of transient IR and Raman spectroscopies for investigating ES-SB. In principle, both techniques should be equivalently powerful to visualise this process. FSRS was applied once previously to study A–D–A systems with a methylated oligosilane core and cyanovinyl-subtituted arene donors.^79^ Only minor spectral dynamics could be observed in the –C=C– and –C=O stretching regions in polar solvents. The authors concluded that ES-SB was faster than the time resolution of the experiment.

As illustrated in Fig. 5, the Raman bands measured here with 1 in the excited state are much broader, 90 cm^−1^ (FWHM) in THF, than the corresponding IR bands, 25–35 cm^−1^. Because of this, the rise of a weaker nearby band resulting from ES-SB cannot be resolved in the Raman spectrum, while it can be easily observed in the IR spectrum. Consequently, the occurrence of symmetry breaking in 1 cannot be concluded solely from the FSRS data.

This problem is not due to the spectral resolution of the FSRS setup, which amounts to *ca.* 10 cm^−1^. This is sufficient to resolve the two –C≡C– stretching bands of 1 in THF that are approx. 65 cm^−1^ apart. The large Raman bandwidth is most probably related to the electronic resonance enhancement process. According to the theory of resonance Raman spectroscopy and FSRS,^80,81^ the width of the Raman lines is related to the lifetime of the electronic excited states involved in the resonance. The FSRS measurements with 1 were done in resonance with a S_*n*>1_ ← S_1_ transition. The large Raman bandwidth observed here is therefore consistent with the very short lifetime of the S_*n*>1_ state. One way to circumvent this problem would be to probe either far from resonance or in resonance with the S_1_ → S_0_ transition. The solubility of 1 is too weak to reach the concentrations required for non-resonant measurements. Additionally, attempts to obtain FSRS spectra of 1 with the Raman pulses in the stimulated emission region did not succeed.

## 5 Conclusions

We compared the excited-state properties of two A–D–A dyes that differ by the nature of the accepting groups, cyanophenyl (1) *vs.* dicyanovinyl (2). Although the former is a weaker electron attracting group than the latter, ES-SB is observed with the cyanophenyl-based dye already in weakly polar environments, whereas the excited state of the other dye remains essentially symmetric even in highly polar solvents. These results highlight the key importance of the coupling between the two A–D branches, *V*_ib_, on the propensity of a quadrupolar dye to undergo ES-SB and to become dipolar. For symmetry to be broken, the loss of this coupling energy should be compensated by a gain in solvation energy. For 2, the coupling is so large that it can never be counterbalanced by solvation. This high *V*_ib_ is most probably due to the small size of the branches. Although cyanophenyl is a weaker electron attracting group, ES-SB takes place with 1 because of the significantly weaker interbranch coupling, arising from longer D–A branches. However, comparison of dye 1 with other cyanophenyl-based A–D–A dyes containing different linkers reveals that larger D–A distances do not warrant smaller coupling, especially when ethynyl groups are used as spacers. As both *V*_ib_ and the solvation energy of the excited state are accessible from stationary absorption and emission measurements, symmetry breaking can, in principle, be easily predicted. However, unambiguous evidence for this process in dyes that have vibrational marker groups in the branches can be obtained by time-resolved vibrational spectroscopy. Here we compared for the first time, to the best of our knowledge, transient IR and Raman spectroscopies for visualising ES-SB. The rise of new bands accompanying this process could be clearly detected in the IR spectra, but not in the Raman spectra because of their too large width. This broadening of the FSRS bands is probably due to the resonance enhancement with a transition to an upper-excited electronic state with a very short lifetime. However, exploiting other resonances with longer-lived excited states should certainly make FSRS, in combination with transient IR, a powerful spectroscopic tool to investigate ES-SB.

## Conflicts of interest

There are no conflicts to declare.

## Supplementary Material

CP-025-D3CP02810K-s001
