# Supplementary material for: Excited-state symmetry breaking in quadrupolar pull–push–pull molecules: dicyanovinyl vs. cyanophenyl acceptors
Source: Phys Chem Chem Phys. 2023 Aug 2;25(34):22689–99. doi: 10.1039/d3cp02810k (PMC10467566; doi:10.1039/d3cp02810k)
Supplement: CP-025-D3CP02810K-s001 [file CP-025-D3CP02810K-s001.pdf]

Electronic Supplementary Material (ESI) for Physical Chemistry Chemical Physics.
This journal is © the Owner Societies 2023

Excited-state symmetry breaking in quadrupolar pull-push-pull molecules: dicyanovinyl vs. cyanophenyl acceptors

Pragya Verma[a], Mariusz Tasior[b], Palas Roy[c], Stephen R. Meech*[c], Daniel T. Gryko[b], and Eric Vauthey*[a]

[a] *Department of Physical Chemistry, University of Geneva, 30 Quai Ernest-Ansermet, CH-1211 Geneva 4, Switzerland. E-mail: eric.vauthey@unige.ch*
[b] *Institute of Organic Chemistry, Polish Academy of Sciences, 01-224 Warsaw, Poland*
[c] *School of Chemistry, University of East Anglia, Norwich Research Park, Norwich NR4 7TJ, U.K.*

Contents

S1 Stationary electronic spectroscopy 4

S2 Time-correlated single photon counting 5

S3 Electronic transient absorption spectroscopy 6

S4 Stationary and time-resolved IR spectroscopy 9

S5 Femtosecond stimulated Raman spectroscopy (FSRS) 14

S6 Quantum-chemical calculations 16

List of Figures

S1 Maximum of the $S_1 \leftarrow S_0$ absorption band of dyes **1** and **2** as a function of the electronic (left) and orientational (right) components of the solvent polarisation. ACN: acetonitrile; CHX: cyclohexane; DCM: dichloromethane; DMSO: dimethylsulfoxide; THF: tetrahydrofuran; TOL: toluene. . . 4

S2 Maximum of the $S_1 \rightarrow S_0$ emission band of dyes **1** and **2** as a function of the orientational component of the solvent polarisation, and slopes of the best linear fit. 4

S3 Time dependence of the fluorescence intensity measured by TCSPC upon 395 nm excitation of **1** in different solvents and best fits of the convolution of a single exponential with the instrument response function (IRF). The resulting lifetimes are given in the legend. 5

S4 Transient electronic absorption spectra recorded at various time delays after excitation of dye **1** in cyclohexane at 430 nm (top) and chloroform (middle) and THF at 400 nm (bottom). The negative stationary absorption and stimulated emission spectra are shown in grey. 6

S5 Evolution-associated difference absorption spectra (EADS) and time constants obtained from a global analysis of the transient electronic absorption spectra recorded with dye **1** assuming a series of exponential steps with increasing time constants. The time constant associated with the D→E step corresponds to the excited-state lifetime. 7

S6 Transient electronic absorption spectra recorded at various time delays after excitation of dye **2** in THF at 510 nm (top) and in a PVB film at 430 nm (bottom). The negative stationary absorption and stimulated emission spectra are shown in grey. 8

S7 Evolution-associated difference absorption spectra (EADS) and time constants obtained from a global analysis of the transient electronic absorption spectra recorded with dye **2** assuming a series of exponential steps with increasing time constants. The time constant associated with the C→D step corresponds to the excited-state lifetime. 9

S8 Stationary IR absorption spectra dyes **1** and **2** in chloroform. 9

S9 Transient IR absorption spectra recorded at various time delays after 430 nm excitation of dye **1** in toluene and chloroform. 10

S10 Evolution-associated difference absorption spectra (EADS) and time constants obtained from a global analysis of the transient IR absorption spectra recorded with dye **1** assuming a series of exponential steps with increasing time constants. 11

S11 Transient IR absorption spectra recorded at various time delays after 480 nm excitation of dye **2** in THF and 2-chloroethanol. 12

S12 Evolution-associated difference absorption spectra (EADS) and time constants obtained from a global analysis of the transient IR absorption spectra recorded with dye **2** assuming a series of exponential steps with increasing time constants. 13

S13 Baseline correction of raw FSRS signal of dye-1 in THF. 15

S14 Comparison of the frontier molecular orbitals of dye **1** and its dipolar analogue **1d**. For both dyes, the S_1 state is associated with a one-electron HOMO-LUMO transition. 16

S15 Comparison of the frontier molecular orbitals of dye **2** and its dipolar analogue **2d**. For both dyes, the S_1 state is associated with a one-electron HOMO-LUMO transition. 18

S16 Energies of the first singlet excited states of dyes **1** and **2** determined from TD-DFT calculations. The oscillator strengths, *f*, are for one-photon transitions. 19

List of Tables

S1 Quantum-chemical calculations of the symmetric (s) and antisymmetric (a) stretching frequencies (in cm^{-1}) of **1** and its dipolar analogue (**1d**) in the S_0 and S_1 states. The IR intensities (in km/mol) and Raman intensities (in $Å^4$/AMU) are in given between brackets in red and blue. The changes observed by going from **1**(S_1) to **1d**(S_1) should be equivalent to those occurring upon partial ES-SB. 17

S2 Quantum-chemical calculations of the symmetric (s) and antisymmetric (a) stretching frequencies (in cm^{-1}) of **2**, **2** with an electric dipole field of 1 GV/m, and the dipolar analogue (**2d**). The IR intensities (in km/mol) are in given between brackets. 'In' and 'out' designate the in-phase and out-of-phase vibrations. The changes observed by going from **2**(S_1) to **2**(S_1)+field should be equivalent to those occurring upon partial ES-SB. The changes observed by going from **2**(S_1) to **2d**(S_1) should be equivalent to those occurring upon full ES-SB. 17

S1 Stationary electronic spectroscopy

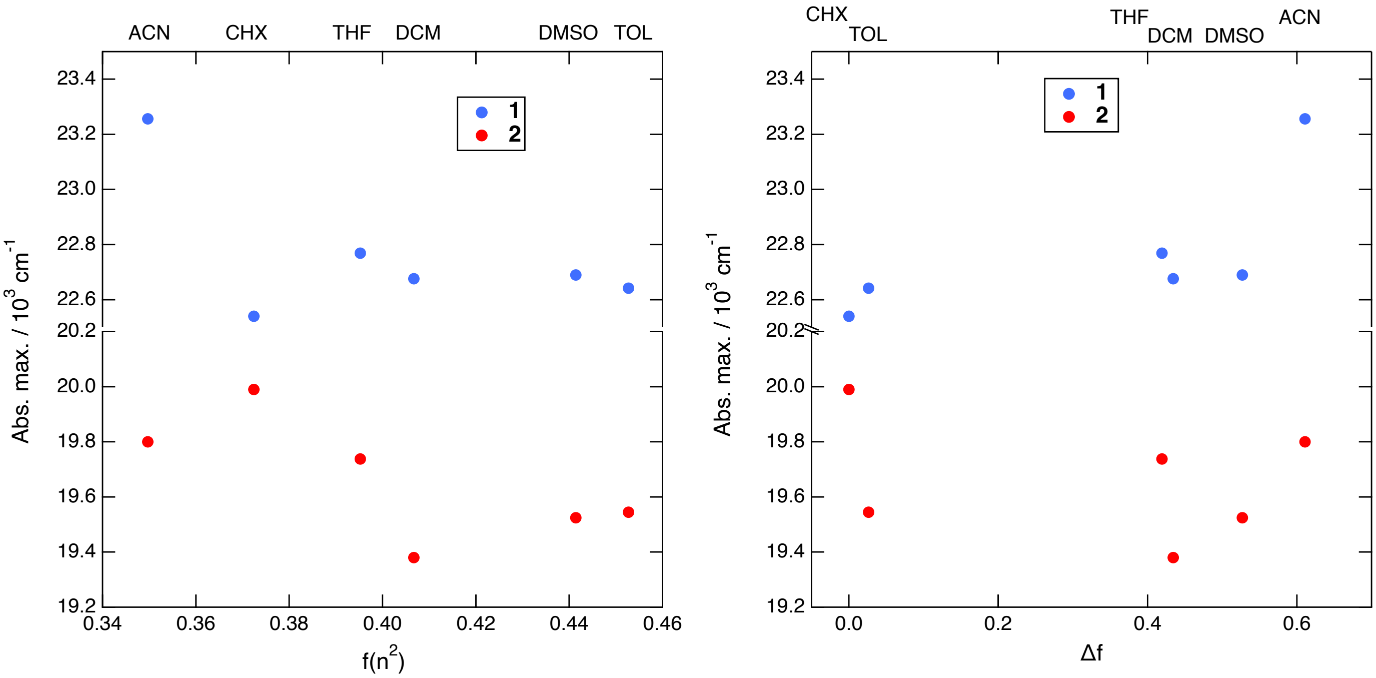

Figure S1 Maximum of the $S_1 \leftarrow S_0$ absorption band of dyes **1** and **2** as a function of the electronic (left) and orientational (right) components of the solvent polarisation. ACN: acetonitrile; CHX: cyclohexane; DCM: dichloromethane; DMSO: dimethylsulfoxide; THF: tetrahydrofuran; TOL: toluene.

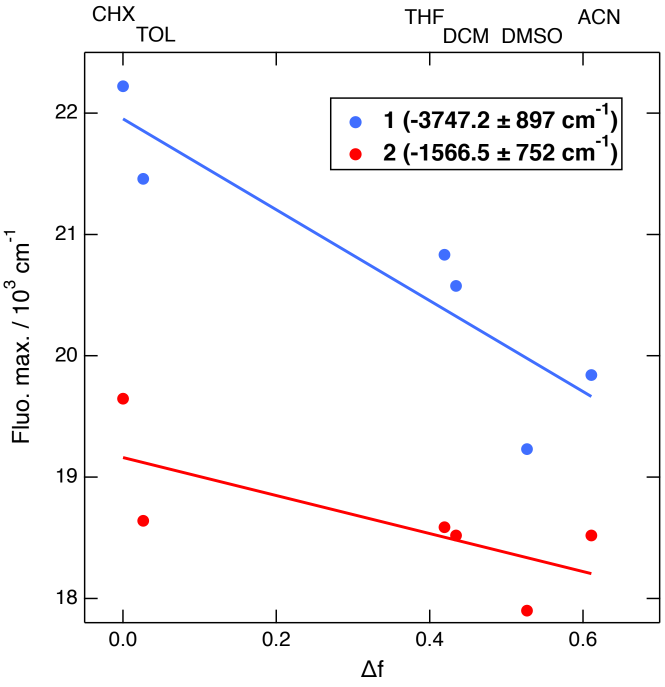

Figure S2 Maximum of the $S_1 \rightarrow S_0$ emission band of dyes **1** and **2** as a function of the orientational component of the solvent polarisation, and slopes of the best linear fit.

S2 Time-correlated single photon counting

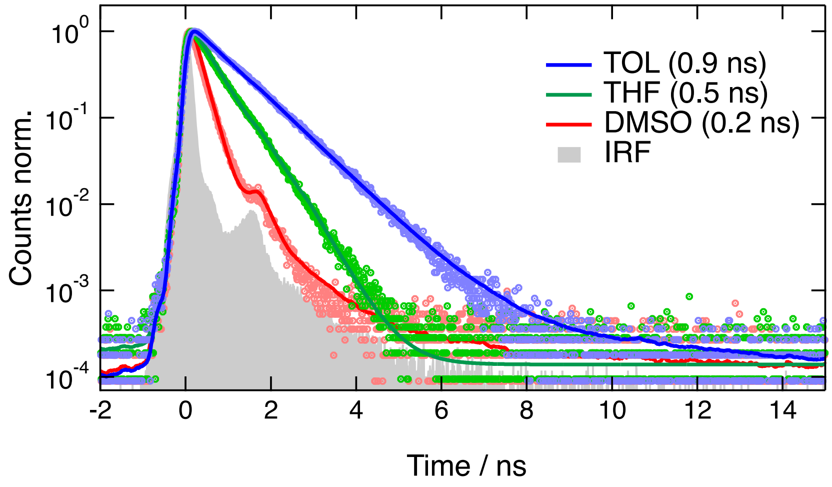

Figure S3 Time dependence of the fluorescence intensity measured by TCSPC upon 395 nm excitation of **1** in different solvents and best fits of the convolution of a single exponential with the instrument response function (IRF). The resulting lifetimes are given in the legend.

S3 Electronic transient absorption spectroscopy

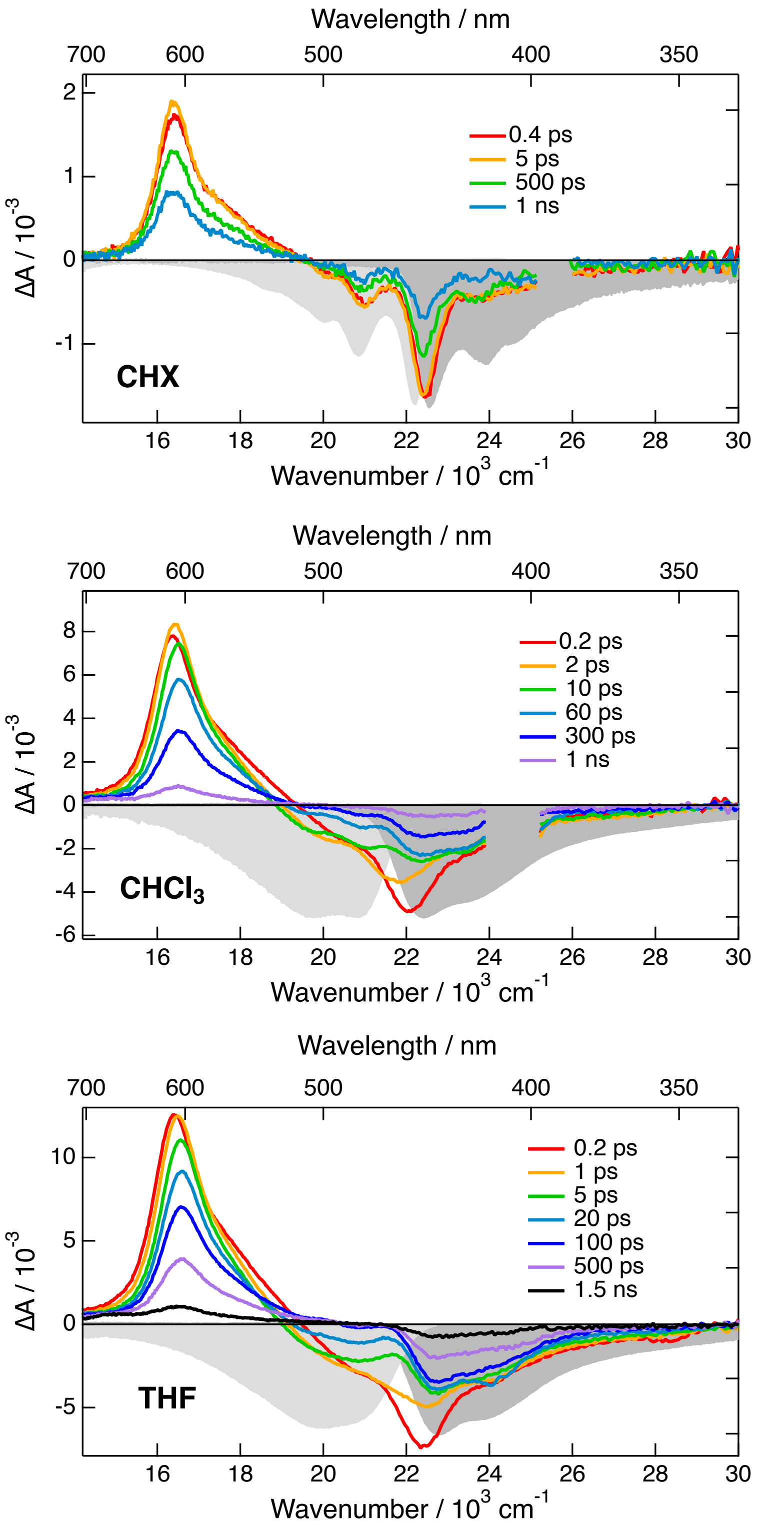

Figure S4 Transient electronic absorption spectra recorded at various time delays after excitation of dye **1** in cyclohexane at 430 nm (top) and chloroform (middle) and THF at 400 nm (bottom). The negative stationary absorption and stimulated emission spectra are shown in grey.

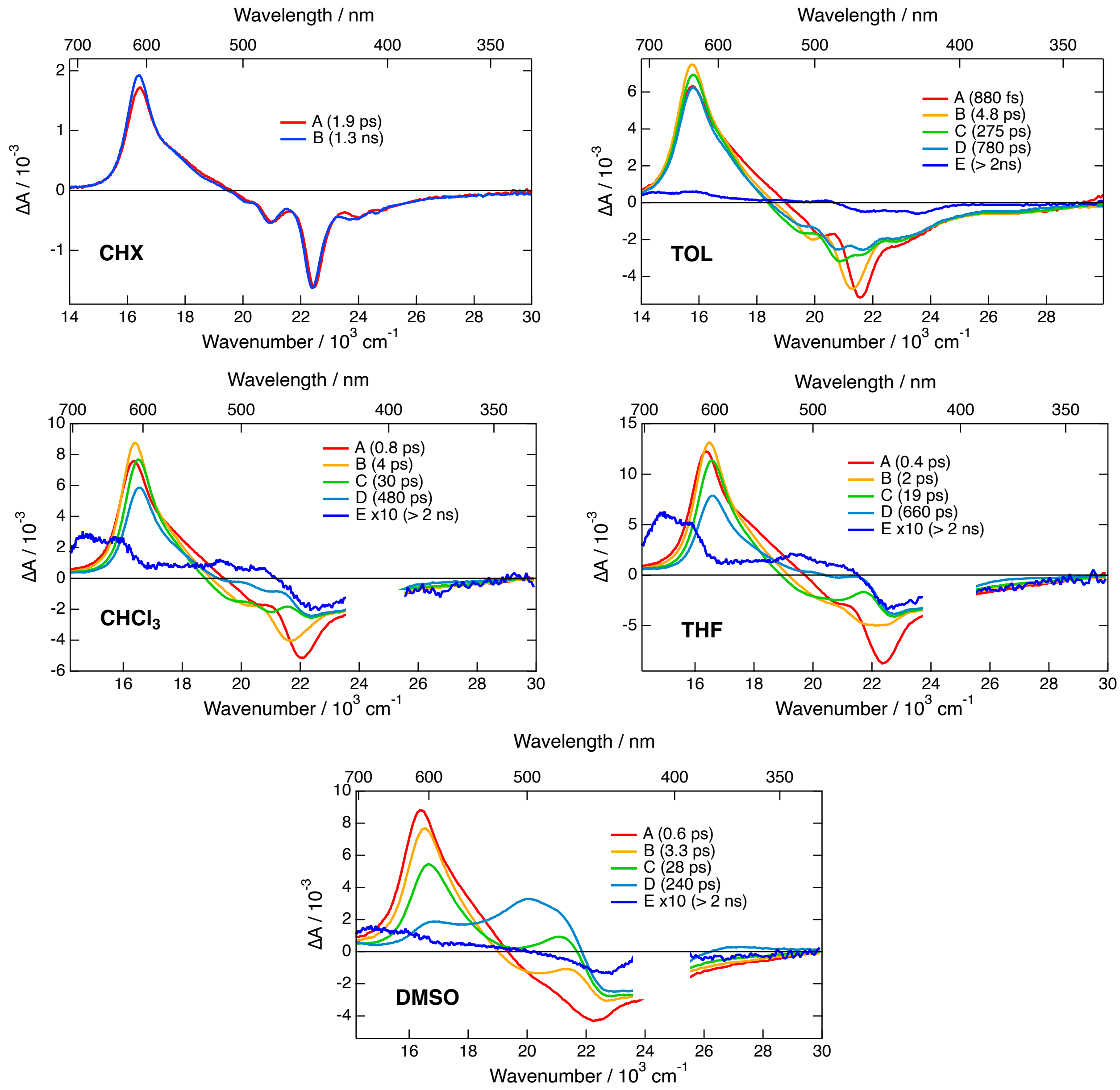

Figure S5 Evolution-associated difference absorption spectra (EADS) and time constants obtained from a global analysis of the transient electronic absorption spectra recorded with dye **1** assuming a series of exponential steps with increasing time constants. The time constant associated with the D$\rightarrow$E step corresponds to the excited-state lifetime.

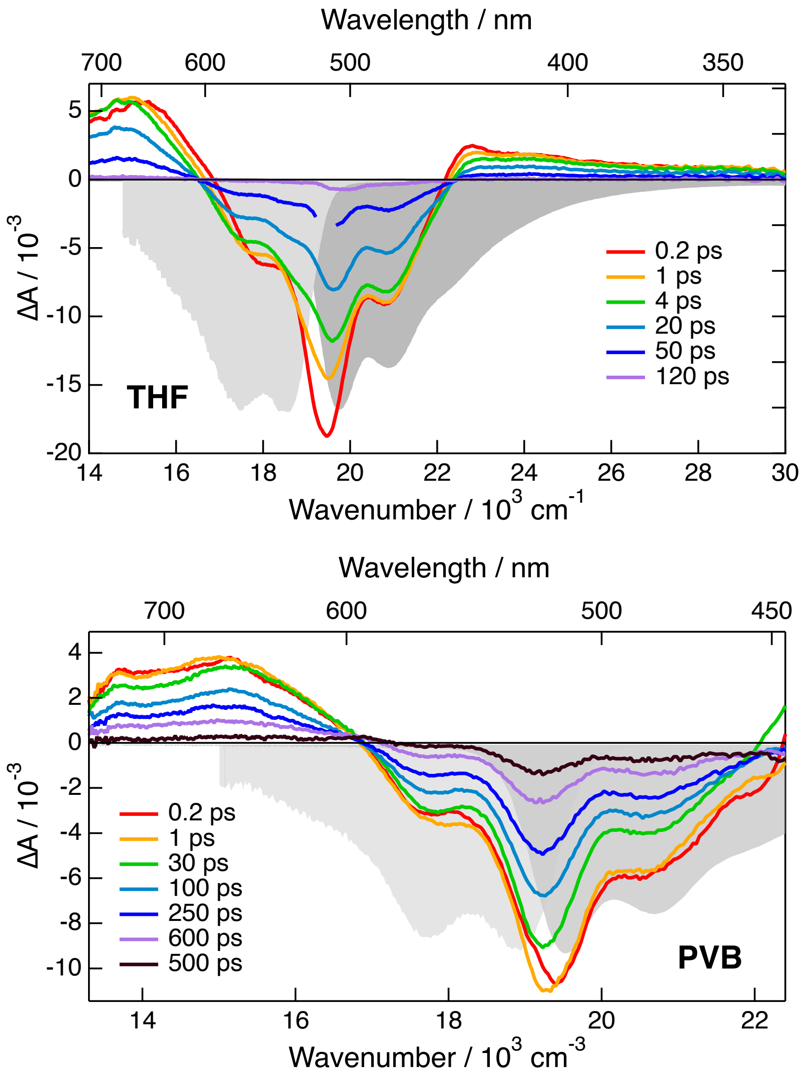

Figure S6 Transient electronic absorption spectra recorded at various time delays after excitation of dye **2** in THF at 510 nm (top) and in a PVB film at 430 nm (bottom). The negative stationary absorption and stimulated emission spectra are shown in grey.

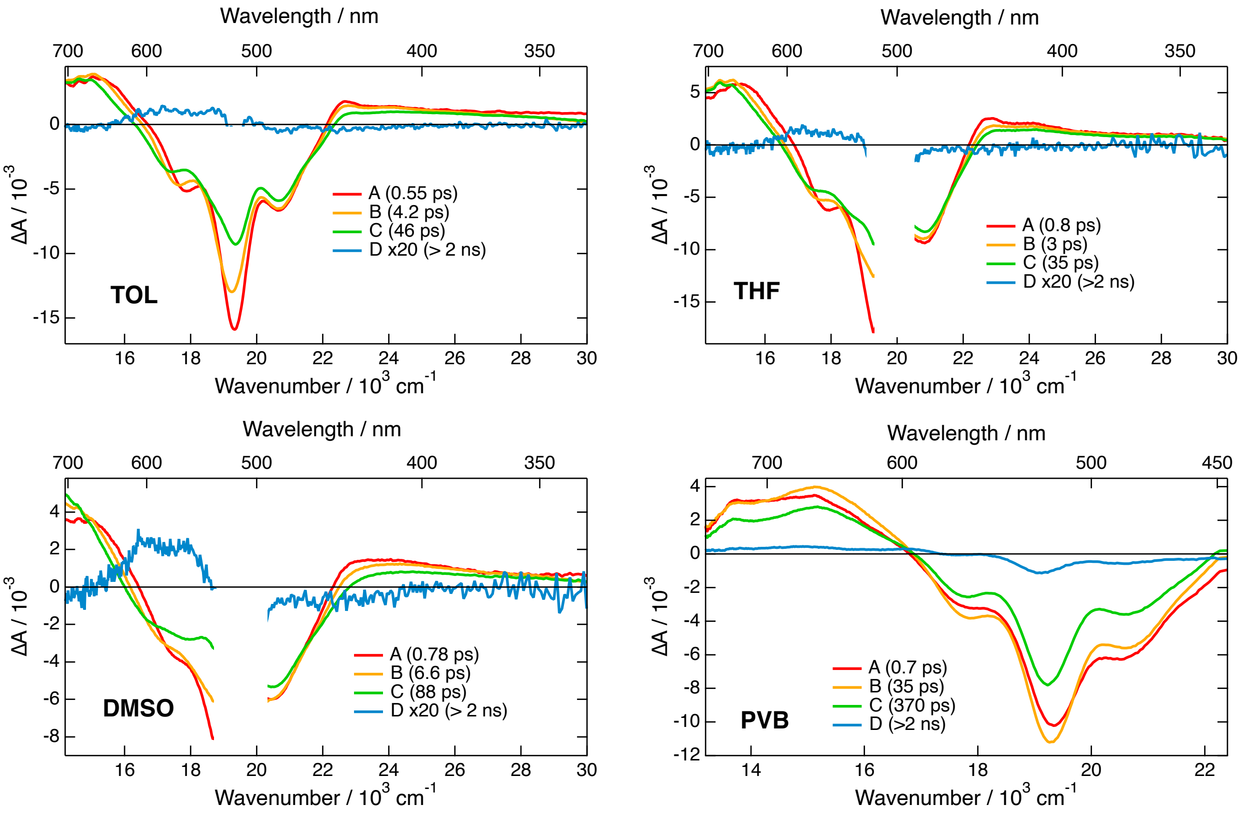

Figure S7 Evolution-associated difference absorption spectra (EADS) and time constants obtained from a global analysis of the transient electronic absorption spectra recorded with dye **2** assuming a series of exponential steps with increasing time constants. The time constant associated with the C→D step corresponds to the excited-state lifetime.

S4 Stationary and time-resolved IR spectroscopy

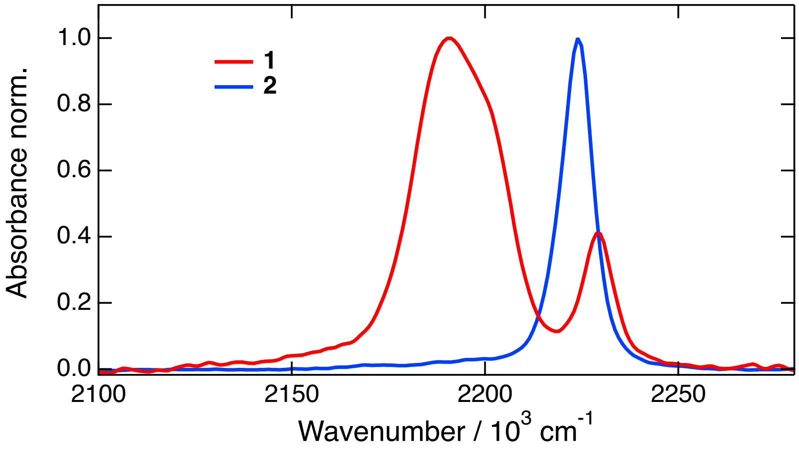

Figure S8 Stationary IR absorption spectra dyes **1** and **2** in chloroform.

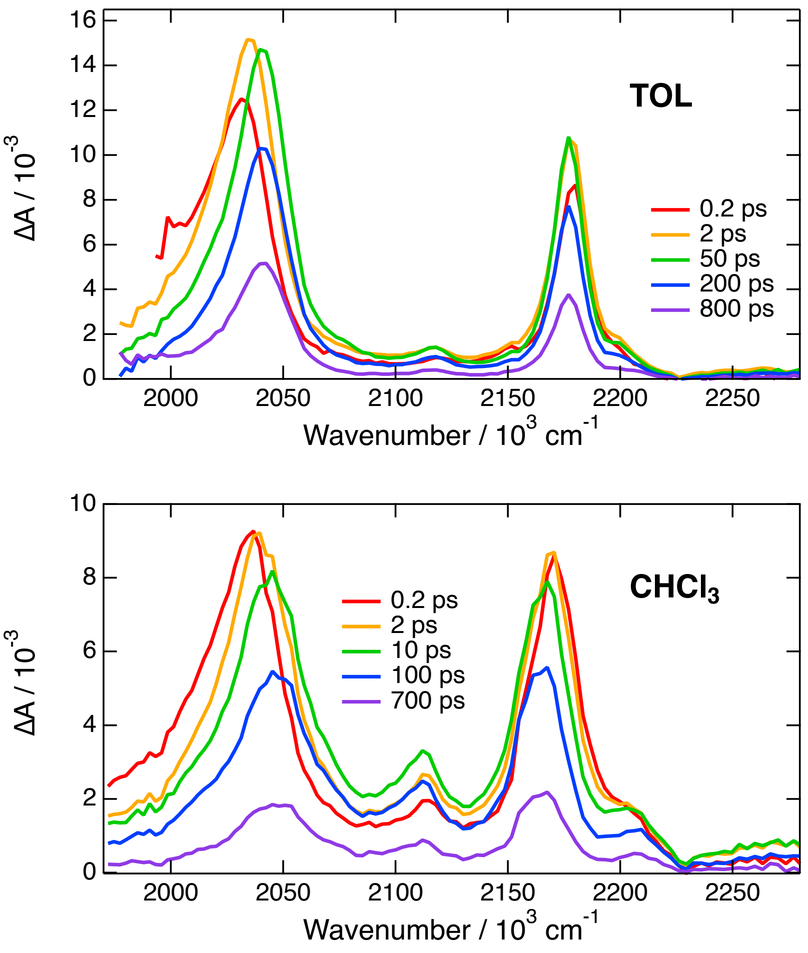

Figure S9 Transient IR absorption spectra recorded at various time delays after 430 nm excitation of dye **1** in toluene and chloroform.

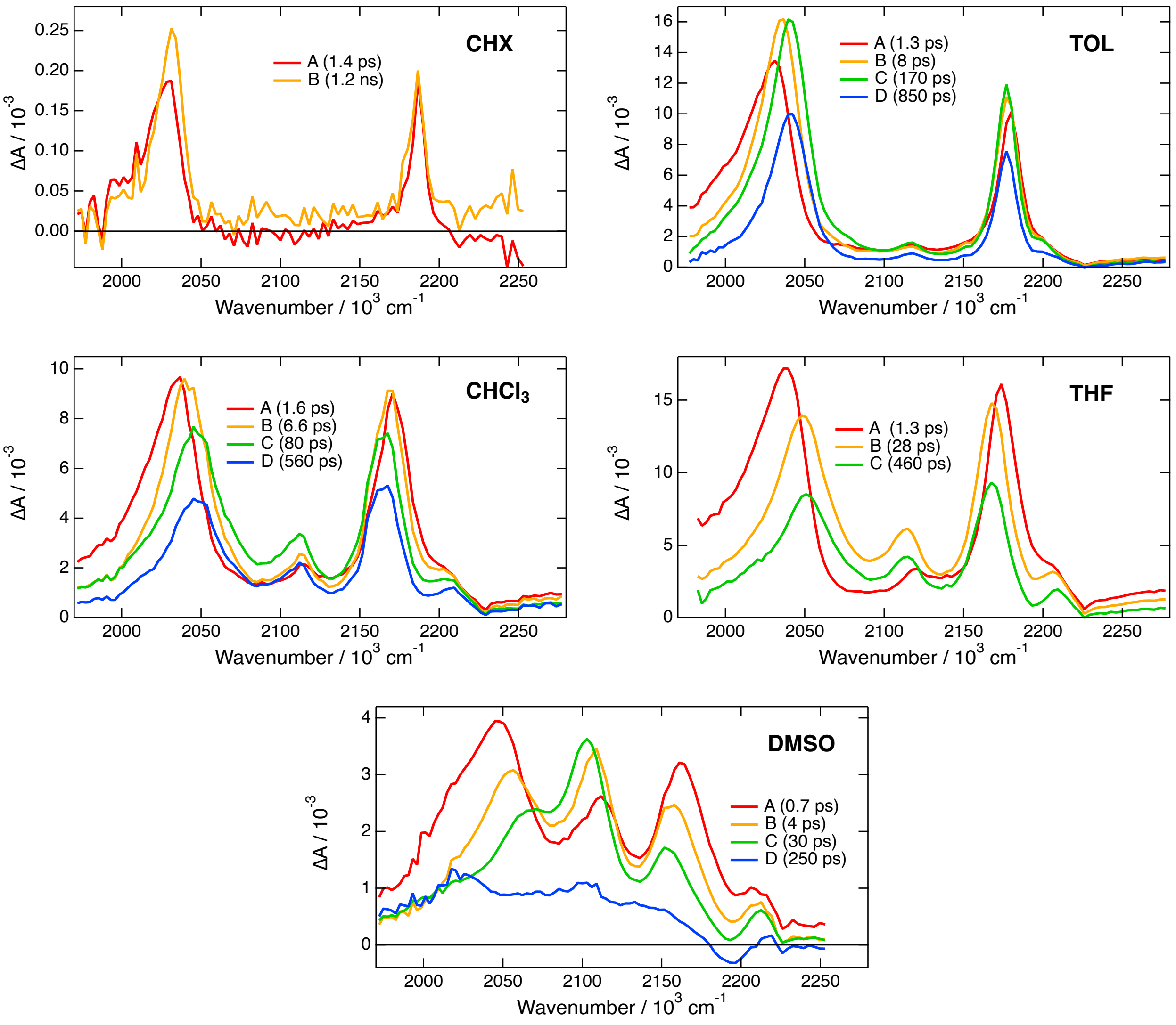

Figure S10 Evolution-associated difference absorption spectra (EADS) and time constants obtained from a global analysis of the transient IR absorption spectra recorded with dye **1** assuming a series of exponential steps with increasing time constants.

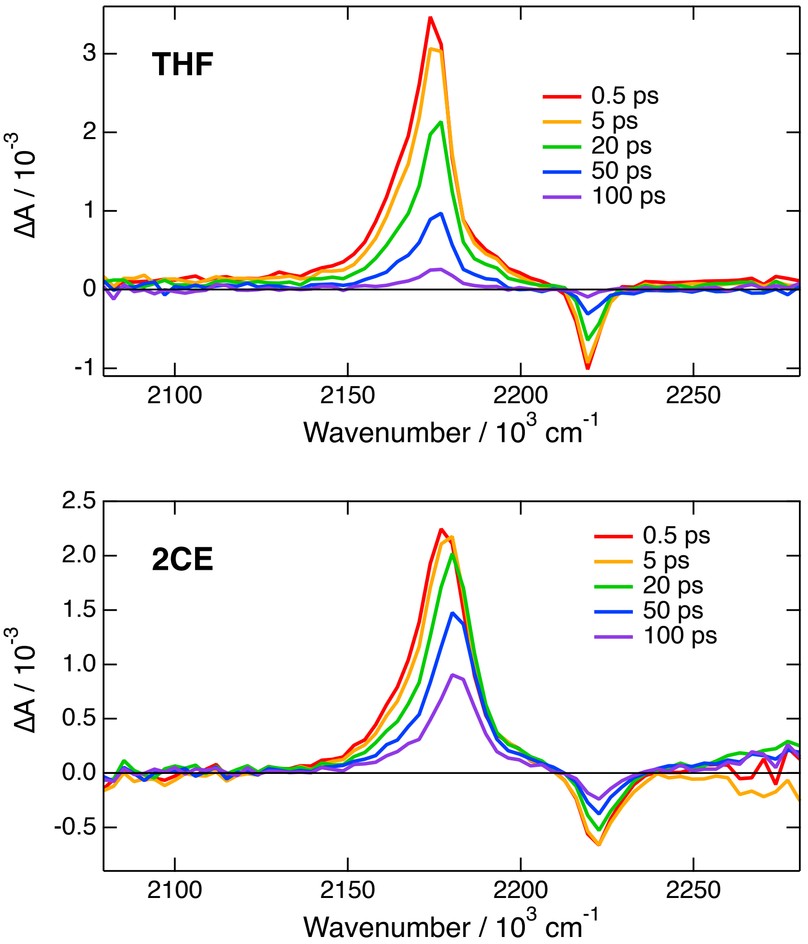

Figure S11 Transient IR absorption spectra recorded at various time delays after 480 nm excitation of dye **2** in THF and 2-chloroethanol.

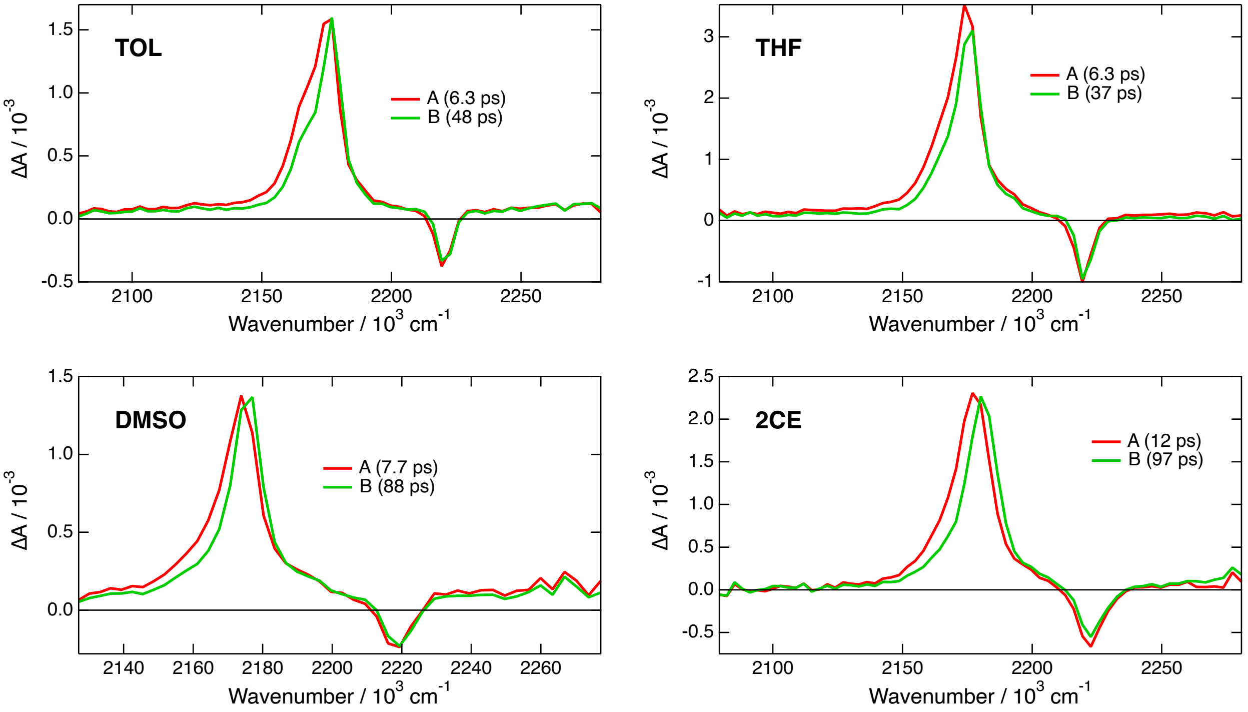

Figure S12 Evolution-associated difference absorption spectra (EADS) and time constants obtained from a global analysis of the transient IR absorption spectra recorded with dye **2** assuming a series of exponential steps with increasing time constants.

S5 Femtosecond stimulated Raman spectroscopy (FSRS)

Details on the FSRS set up used here have been presented elsewhere.[1] Briefly, FSRS is a three pulse technique with femtosecond actinic pump, femtosecond white-light probe and picosecond Raman pump pulses. The actinic pump excites the molecule in its electronic ground state to the higher excited state. Then a broadband femtosecond probe pulse in the presence of a narrowband (10 cm^{-1}) picosecond Raman pulse stimulate the coherent Raman scattering process from the sample. These pulses were generated from the fundamental output of a regenerative Ti:sapphire amplifier (Spectra Physics Spitfire ACE) driven by a Spectra Physics Mai Tai laser oscillator. Part of this amplified pulse centered at 800 nm with repetition rate of 1 kHz, duration of 120 fs, and energy of 5 mJ per pulse was used to drive a commercial optical parametric amplifier (OPA, Light Conversion TOPAS Prime). This OPA generated the tuneable actinic pump pulse used to excite the sample. Another part of the fundamental 800 nm beam was used to drive a second OPA which generated pulses at 1250 nm. This 1250 nm pulse was focused on to a 3 mm thick sapphire window to generate broadband white light continuum (500-1400 nm). The rest of the amplified fundamental beam was directed through a commercial second harmonic bandwidth compressor (SHBC from Light Conversion) and then a picosecond configured OPA (TOPAS-PS from Light Conversion) to generate the tuneable picosecond Raman pump.

The actinic pump, Raman pump and continuum probe pulses were focused and overlapped spatially and temporally inside a 1 mm pathlength sample cell. Their spot sizes were adjusted to be around 250, 150 and 50 μm respectively. The transmitted probe beam was dispersed using a high spectral resolution ($<$10 cm^{-1}) grating spectrometer (SPEX 500M) and then detected using a single CCD (Entwicklungsbüro Stresing, 1024 pixel). The stimulated Raman signal from cyclohexane was used to calibrate the detector and overlap the probe and Raman pulses. Line width of the Raman peak of cyclohexane at 802 cm^{-1} was used to determine experimental spectral resolution, which is about 10 cm-1. Time delay between the actinic pump and probe pulses was generated using a computer-controlled delay stage. Each spectrum was accumulated for 6 s using a LabView controlled software. The actinic pump power used was 0.2 mW at 430 nm. The Raman pulse was tuned at 620 nm (2 mW) to be in resonance with the excited state absorption. Solvent response signal in the presence of the actinic pump and probe pulses was used to determine time resolution of the experiment, which is about 100 fs. All the spectra were recorded at 1 kHz. The actinic pump and Raman pump pulses were passed through two synchronized mechanical choppers operating at 500 Hz and 250 Hz respectively. This resulted in four different signals being measured: i) Raw FSRS (Probe + Raman + Actinic); ii) Transient absorption (Probe + Actinic); iii) Ground state FSRS (Probe + Raman) and iv) Probe reference (probe only). When all the three pulse (Actinic pump, Raman pump and Probe) are present on the sample, we detect the unprocessed FSRS signal as $\log(I_{\text{Raman+Actinic+Probe}}/I_{\text{Probe}})$. Therefore, the unprocessed FSRS signal contains information on the excited state Raman spectra along with the ground state Raman ($\log(I_{\text{Raman+Probe}}/I_{\text{Probe}})$) , transient absorption (TA) ($\log(I_{\text{Actinic+Probe}}/I_{\text{Probe}})$) and nonlinear background. Therefore, the unprocessed FSRS data has been subtracted from its ground state Raman and TA contributions in order to extract the excited state Raman signal. Then the resultant difference spectra (say, raw excited state Raman signal) are baseline corrected to obtain the raw excited state Raman signal as shown in Figure S13. This same procedure is followed for all the pump-probe time delays and for all the solvents.

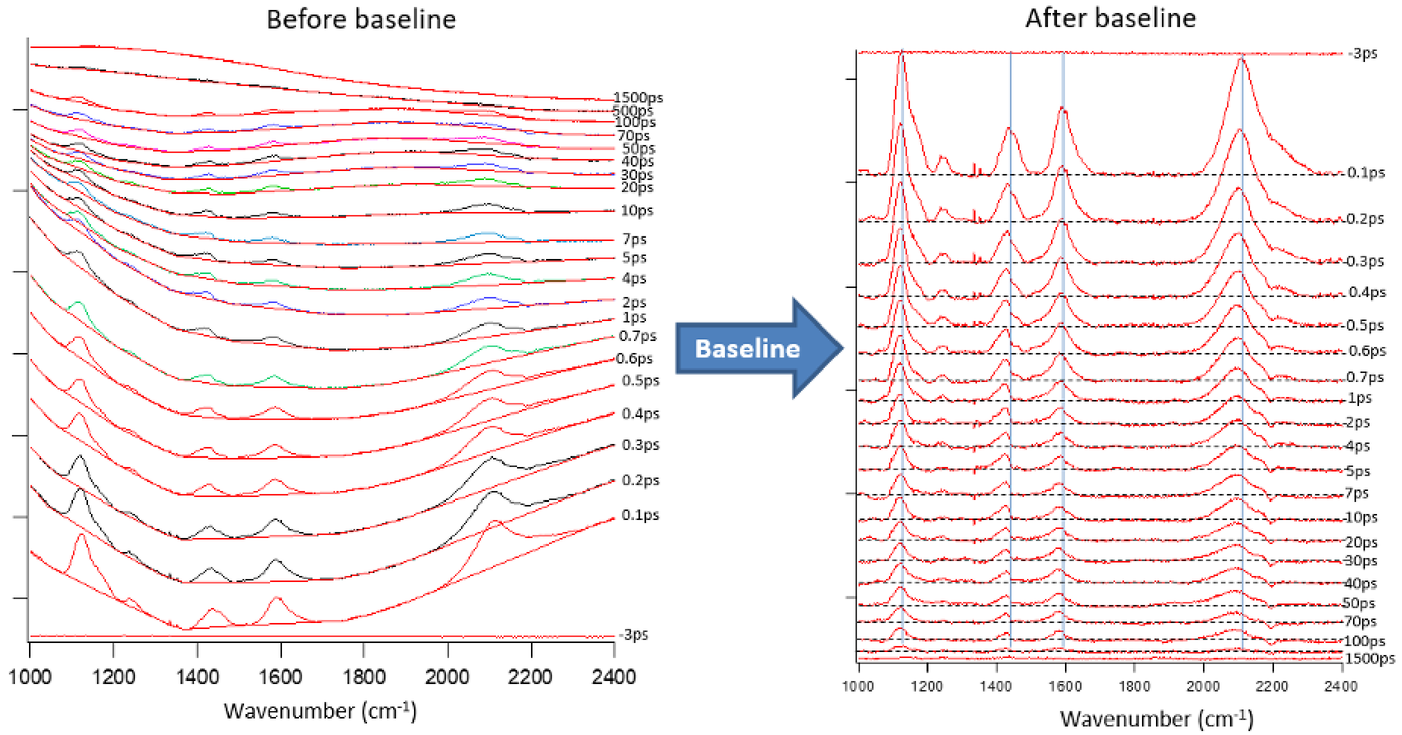

Figure S13 Baseline correction of raw FSRS signal of dye-1 in THF.

S6 Quantum-chemical calculations

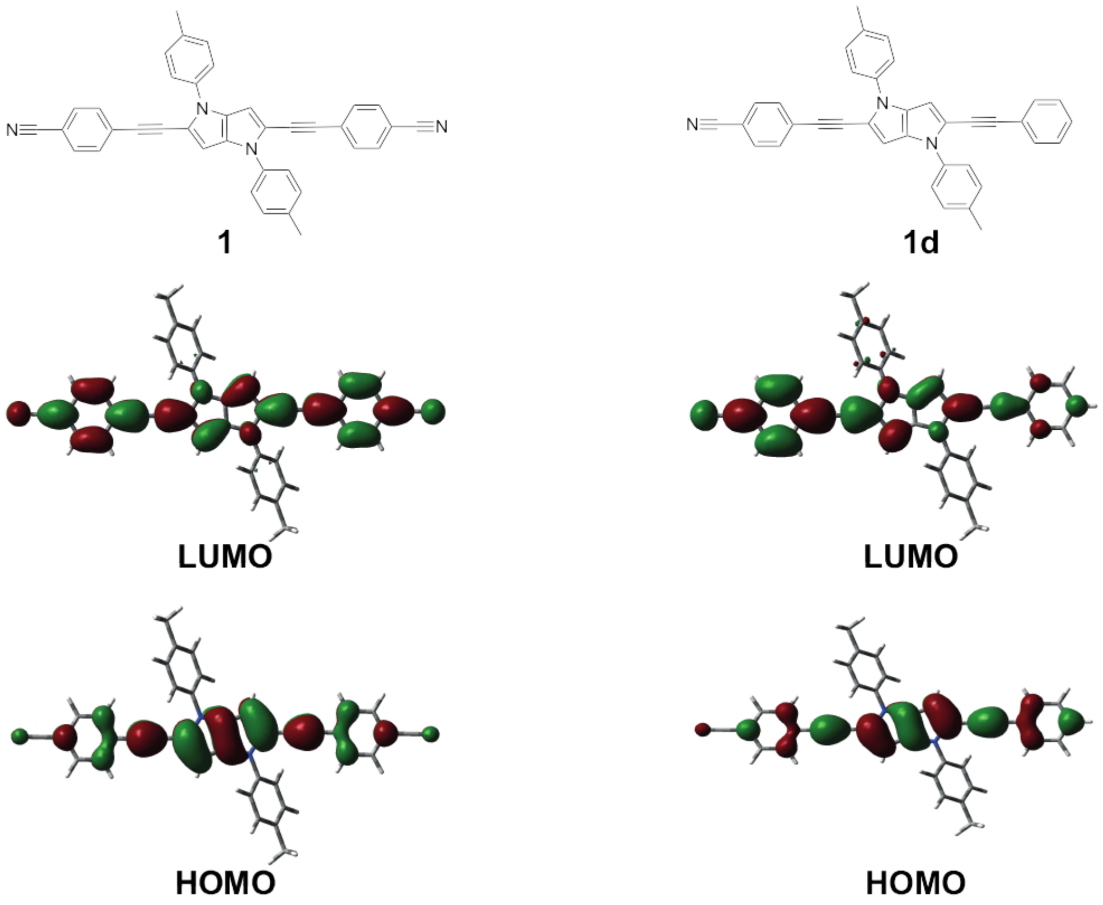

Figure S14 Comparison of the frontier molecular orbitals of dye **1** and its dipolar analogue **1d**. For both dyes, the S_1 state is associated with a one-electron HOMO-LUMO transition.

Table S1 Quantum-chemical calculations of the symmetric (s) and antisymmetric (a) stretching frequencies (in cm^{-1}) of **1** and its dipolar analogue (**1d**) in the S_0 and S_1 states. The IR intensities (in km/mol) and Raman intensities (in $Å^4$/AMU) are in given between brackets in red and blue. The changes observed by going from **1**(S_1) to **1d**(S_1) should be equivalent to those occurring upon partial ES-SB.

	-C≡C-(s)	-C≡C-(a)	-C≡N(s)	-C≡N(a)
**1**(S_0)	2193 (0, 10^5)	2191 (1274, 0)	2240 (0, 3000)	2240 (134, 0)
**1d**(S_0)	2190 (790, $5.5{\cdot}10^4$)	2200 (110, 3400)		2240 (69, 1500)
**1**(S_1)	2148 (0, 10^6)	1992 (3800, 0)	2220 (0, $1.4{\cdot}10^5$)	2216 (2360, 0)
**1d**(S_1)	2113 (1680, $1.5{\cdot}10^5$)	2006 (350, $8.4{\cdot}10^5$)		2206 (1100, $1.8{\cdot}10^4$)

Table S2 Quantum-chemical calculations of the symmetric (s) and antisymmetric (a) stretching frequencies (in cm^{-1}) of **2**, **2** with an electric dipole field of 1 GV/m, and the dipolar analogue (**2d**). The IR intensities (in km/mol) are in given between brackets. 'In' and 'out' designate the in-phase and out-of-phase vibrations. The changes observed by going from **2**(S_1) to **2**(S_1)+field should be equivalent to those occurring upon partial ES-SB. The changes observed by going from **2**(S_1) to **2d**(S_1) should be equivalent to those occurring upon full ES-SB.

	-C≡N(a, out)	-C≡N(s, out)	-C≡N(a, in)	-C≡N(s, in)
**2**(S_0)	2212 (35)	2212 (0.2)	2220 (440)	2222 (0.0)
**2d**(S_0)	2206 (41)		2216 (325)	
**2**(S_1)	2181 (212)	2185 (1.5)	2202 (560)	2210 (0.2)
**2**(S_1)+field	2175 (90)	2190 (205)	2200 (400)	2213 (67)
**2d**(S_1)	2146 (100)		2181 (170)	

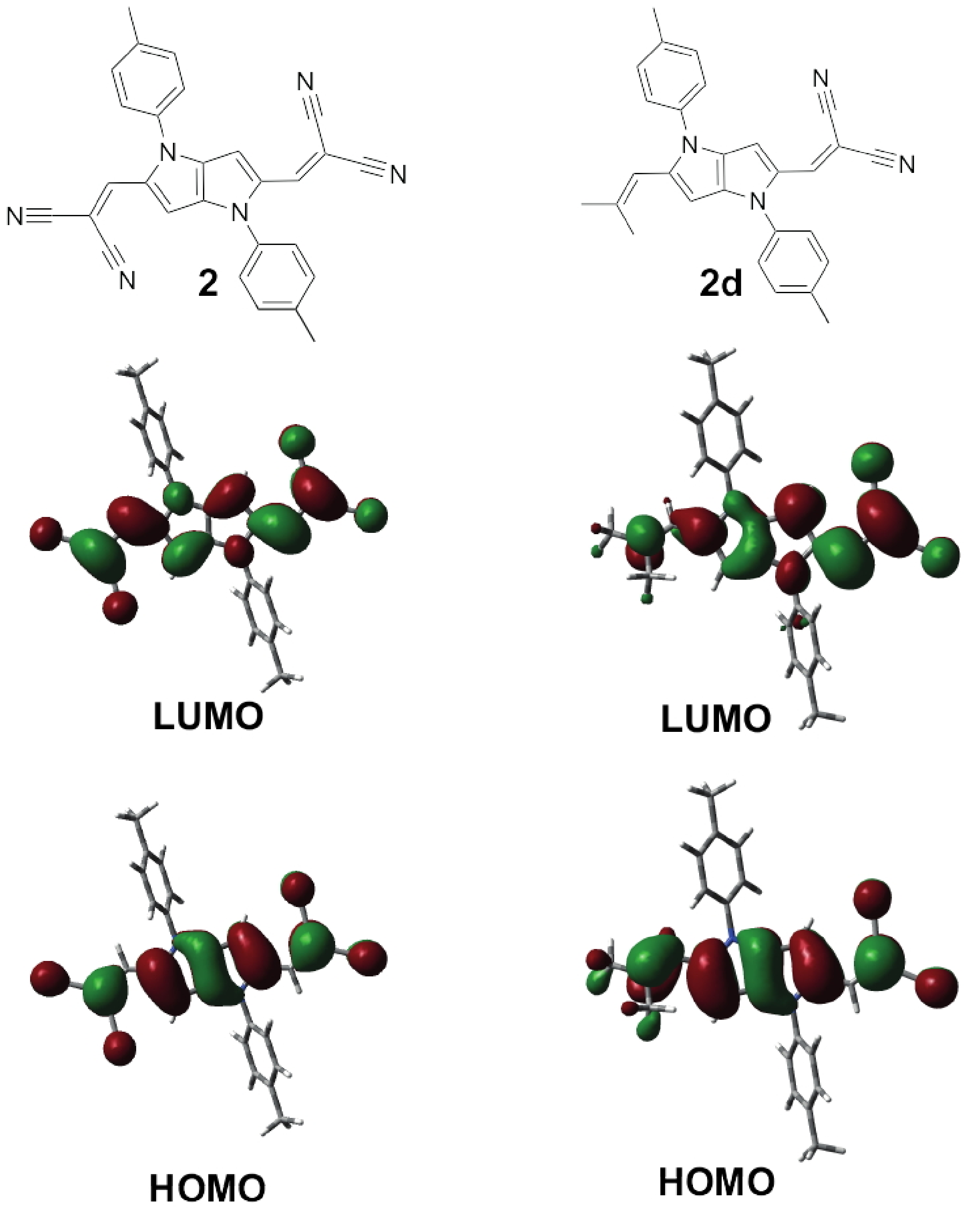

Figure S15 Comparison of the frontier molecular orbitals of dye **2** and its dipolar analogue **2d.** For both dyes, the S_1 state is associated with a one-electron HOMO-LUMO transition.

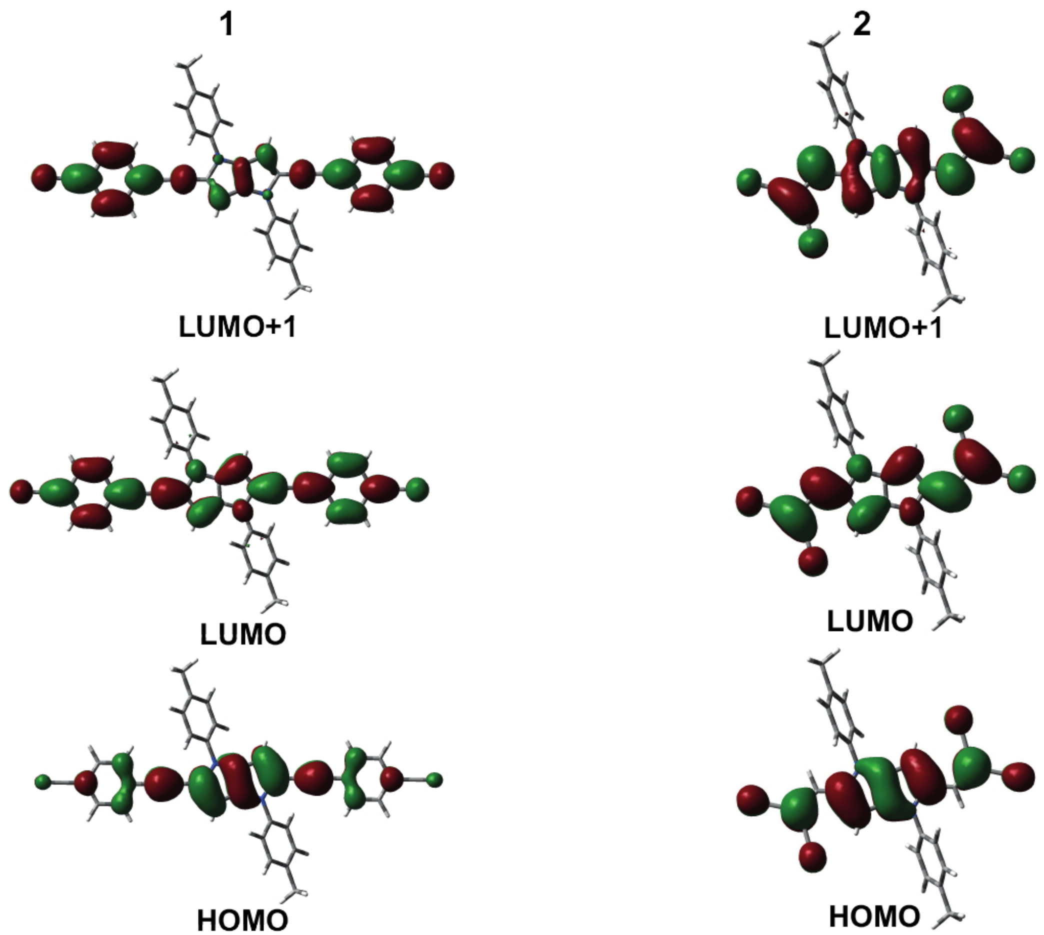

S_1: HOMO-LUMO, E=3.26 eV,
$f(S_1 \leftarrow S_0)$= 2.4

S_2: HOMO-LUMO+1, E=4.09 eV,
$f(S_1 \leftarrow S_0)$=0

S_1: HOMO-LUMO, E=2.99 eV,
$f(S_1 \leftarrow S_0)$= 1.2

S_2: HOMO-1 -LUMO, E=3.39 eV,
$f(S_2 \leftarrow S_0)$=0.3

S_3: HOMO-LUMO+1, E=4.24 eV,
$f(S_3 \leftarrow S_0)$=0

Figure S16 Energies of the first singlet excited states of dyes **1** and **2** determined from TD-DFT calculations. The oscillator strengths, *f*, are for one-photon transitions.

References

[1] P. Roy, W. R. Browne, B. L. Feringa and S. R. Meech, *Nature Commun.*, 2023, **14**, 1253.
